# Metabolic Biomarkers of Ageing in C57BL/6J Wild-Type and Flavin-Containing Monooxygenase 5 (FMO5)-Knockout Mice

**DOI:** 10.3389/fmolb.2018.00028

**Published:** 2018-04-09

**Authors:** Dorsa Varshavi, Flora H. Scott, Dorna Varshavi, Sunil Veeravalli, Ian R. Phillips, Kirill Veselkov, Nicole Strittmatter, Zoltan Takats, Elizabeth A. Shephard, Jeremy R. Everett

**Affiliations:** ^1^Medway Metabonomics Research Group, University of Greenwich, Chatham, United Kingdom; ^2^Institute of Structural and Molecular Biology, University College London, London, United Kingdom; ^3^School of Biological and Chemical Sciences, Queen Mary University of London, London, United Kingdom; ^4^Department of Surgery and Cancer, Faculty of Medicine, Imperial College, London, United Kingdom

**Keywords:** metabonomics, metabolomics, ageing, C57BL/6J, FMO5 KO, urine, plasma, 6-hydroxy-6-methyl-heptan-3-one

## Abstract

It was recently demonstrated in mice that knockout of the flavin-containing monooxygenase 5 gene, *Fmo5*, slows metabolic ageing via pleiotropic effects. We have now used an NMR-based metabonomics approach to study the effects of ageing directly on the metabolic profiles of urine and plasma from male, wild-type C57BL/6J and *Fmo5*^−/−^ (FMO5 KO) mice back-crossed onto the C57BL/6J background. The aim of this study was to identify metabolic signatures that are associated with ageing in both these mouse lines and to characterize the age-related differences in the metabolite profiles between the FMO5 KO mice and their wild-type counterparts at equivalent time points. We identified a range of age-related biomarkers in both urine and plasma. Some metabolites, including urinary 6-hydroxy-6-methylheptan-3-one (6H6MH3O), a mouse sex pheromone, showed similar patterns of changes with age, regardless of genetic background. Others, however, were altered only in the FMO5 KO, or only in the wild-type mice, indicating the impact of genetic modifications on mouse ageing. Elevated concentrations of urinary taurine represent a distinctive, ageing-related change observed only in wild-type mice.

## Introduction

The ageing of the human population represents a huge, current challenge to global healthcare, so much so that it is argued that ageing should be tackled as a disease (Faragher, 2015). Delaying one age-related disease may be associated with beneficially delaying the onset of others (Fontana et al., 2014). However, ageing is a complex biological process that is associated with a number of diseases, such as type-2 diabetes, cardiovascular disease and neurodegeneration (North and Sinclair, 2012; Stoyanova, 2014). In spite of many efforts, the mechanism of ageing is not yet completely understood. The ageing process is characterised by a progressive decline in physiological functional capacity as well as deterioration of metabolic function. The signature of these metabolic changes that occur during the process of maturation and ageing can be investigated using global metabolite profiling (Houtkooper et al., 2011), that is, metabonomics or metabolomics (Lindon et al., 2000).

Ageing can be affected by genetic modification and a number of studies have been performed on long-lived, model mammals, such as growth hormone (GH)/GH receptor-deficient dwarf mice (Coschigano et al., 2003), various insulin/insulin-like growth factor (IGF)-signalling (IIS) pathway (Selman et al., 2011), and mammalian target of rapamycin (TOR)-signalling mutant mice (Selman et al., 2009), in order to identify the mechanisms underlying healthy lifespan and translate this knowledge into practical therapies for humans.

In the present study, a metabonomic, metabolic profiling approach was used to study the effects of ageing in mice in which the gene encoding flavin-containing monooxygenase 5 (FMO5) had been disrupted (*Fmo5*^−/−^) and to compare the ageing profile of these knockout mice (FMO5 KO) with their wild-type (WT) counterparts at equivalent time points. FMO5 is known to be a key regulator of metabolic ageing (Gonzalez Malagon et al., 2015). FMO5 KO mice exhibit both reduced plasma glucose and cholesterol concentrations as they age compared with their WT counterparts (Gonzalez Malagon et al., 2015). In addition, recent studies (Scott et al., 2017) have shown that an absence of FMO5 protein confers the high glucose tolerance and insulin sensitivity associated with young mice onto older mice as they age, and also protects against high-fat diet-induced weight gain and loss of insulin sensitivity. FMO5 was proposed to have a key role in sensing or responding to gut bacteria, with the gut microbiome of FMO5 KO mice being reported as “invisible” to the host mouse (Scott et al., 2017).

The aim of the present study is to identify metabolic signatures that are associated with ageing and to understand the differences in metabolic ageing between WT and FMO5 KO mice. The identification of ageing-associated biomarkers may give a better understanding of the mechanisms of ageing and, thus, provide targets for therapeutic approaches to help extend healthy human lifespans.

## Materials and methods

### Study design

All animals used in this study were male mice bred at University College London (UCL). FMO5 KO mice were obtained after eight generations of backcrosses of heterozygous FMO5 KO mice with WT C57BL/6J mice (Gonzalez Malagon et al., 2015). WT C57BL/6J mice were used as controls. Mice were fed a standard chow diet (Teklad Global 18% Protein Rodent Diet, Harlan Laboratories, Inc., Madison, WI) (Gonzalez Malagon et al., 2015). Animal experiments were carried out in accordance with the UK Animal Procedures Act and with local ethics committee approval (Animal Welfare and Ethical Review Body).

To study the metabolic effects of ageing, blood was collected from the tail vein into heparin-coated tubes and plasma isolated as described. (Hough et al., 2002) Urine samples were collected as described in Kurien et al. (2004) from WT C57BL/6J and FMO5 KO mice, at the ages of 15 (*n* = 4, KO; *n* = 4, WT), 30 (*n* = 4, KO; *n* = 4, WT), 45 (*n* = 5, KO; *n* = 4, WT), and 60 (*n* = 4, KO; *n* = 4, WT) weeks. All samples were collected between 09:00 and 11:00 onto an ice-cooled surface and then frozen on solid CO_2_ and stored at 193 K until analysed by NMR spectroscopy.

The week 15 and week 45 animals were from different cohorts and were each followed longitudinally for 15 weeks. To investigate the effect of batch differences (Li et al., 2013) on the metabolic profiles of urine and plasma, a batch comparison experiment was performed on two different cohorts at the same week 30 time point (week 30 set 2, *n* = 5, KO; *n* = 5, WT, Supplementary Figures 1–4). Since no significant metabolic changes were found between different batches, using ANOVA (Moyé, 2016) with a false discovery rate (FDR) set at 10% (Benjamini, 2010), we proceeded to study the longitudinal effects of ageing on the urinary and plasma metabolic profiles and to compare the profiles of WT and FMO5 KO mice.

### Sample preparation for NMR spectroscopy

Urine samples were prepared by mixing 50 μl of urine from each mouse with 25 μl of phosphate buffer (81:19 (v/v) comprising 0.6 M K_2_HPO_4_ and 0.6 M NaH_2_PO_4_ in 100% ^2^H_2_O, pH 7.4, containing 0.5 mM sodium 3-(trimethylsilyl)-2,2′,3,3′-tetradeuteropropionate (TSP), as a chemical shift reference, and 9 mM sodium azide, as an anti-microbial agent. The buffered samples were then centrifuged (13,000 g for 5 min at 4°C) to remove any suspended particles. After centrifugation, 60 μl of supernatant was transferred into new 1.7 mm outer diameter (o.d.) NMR tubes (Norell, S-1.7-500-1) using an accurate electronic syringe (SGE eVol XR).

Plasma samples were prepared by mixing 50 μl of mouse plasma with 25 μl of saline (0.9% NaCl in ^2^H_2_O which was then centrifuged and 60 μl transferred into new 1.7 mm o.d. microtubes as described above.

Several urine samples and two plasma samples were also prepared as above, but at larger volumes, for analysis in 5 mm o.d. NMR tubes (NORELL, 508-UP-7), in order to provide greater sensitivity for two-dimensional NMR experiments, including 2D ^1^H JRES (J-resolved), COSY (correlated spectroscopy), TOCSY (total correlation spectroscopy), HSQC (heteronuclear single quantum coherence), and HMBC (heteronuclear multiple bond correlation) (Claridge, 2009).

### Ultrafiltration of urine samples for NMR spectroscopic line-width analysis

Urine samples from male, week 12 and week 16 WT and a week 16 FMO5 KO mouse were ultra-filtered by centrifugation at 16,500 g for 20 min at 4°C using a microfilter with a 10 kDa molecular mass cut-off (Merck, UFC501024). Filtered and matching unfiltered samples were then prepared by mixing 40 μl of urine with 20 μl of phosphate buffer (pH 7.4, as above). Aliquots (50 μl) of buffered samples were then transferred into new 1.7 mm o.d. NMR tubes as described above.

The line width of the 6-hydroxy-6-methylheptan-3-one (6H6MH3O) methyl triplet peak at 1.017 ppm was determined by first performing line fitting in MNova 11.0 on the central line, followed by manual half band width measurement. The line width of the 6H6MH3O methyl singlet peak at 1.209 ppm and the peaks due to taurine at 3.433 ppm and trimethylamine at 2.884 ppm were determined by manual half band width measurement. Changes in signal half band width due to the removal of proteins by ultrafiltration were then determined using a 2-tailed student *t*-test, assuming unequal variance, in the StatPlus:mac core v 5.9.50 (AnalystSoft Inc.) plug-in for Excel for Mac 2011 v 14.7.2 (Microsoft Inc.).

### One-dimensional NMR spectroscopic analysis

^1^H NMR spectra of biofluids were recorded on a Bruker Avance III spectrometer (Bruker BioSpin GmbH, Rheinstetten, Germany) operating at 600.44 MHz and at a temperature of 300.0 K.

For urine samples, a standard one-dimensional (1D) NOESY presaturation pulse sequence with gradient pulses (RD-90°-*t*1-90°-*t*m-90°-acquire, Bruker sequence code noesygppr1d) was acquired with water suppression applied during the relaxation delay (RD) of 2 s, a mixing time (tm) of 100 ms and a 90° pulse of 11.2 μs. For each spectrum, 8 dummy scans were used to establish spin equilibrium, then 256 free induction decay transients were collected into 65,536 data points with a spectral width of 20 ppm.

For all mouse plasma samples, two types of 1D ^1^H NMR experiments were acquired. The first was the standard 1D NOESY presaturation pulse sequence (noesygppr1d) with gradient pulses and saturation of the water peak during the relaxation delay (RD). After 8 dummy scans, 128 transients were collected into 65,536 data points over a spectral width of 20 ppm, using a relaxation delay (RD) of 2 s and a mixing time of 100 ms. Standard ^1^H NMR spectra provide information on both low- and high-molecular-mass metabolites. However, signals from low intensity, low-molecular-mass molecules can be obscured by broad signals arising from high-molecular-mass macromolecules. A second set of data was therefore acquired with the Carr–Purcell–Meiboom–Gill (CPMG) spin-echo experiment, RD [90°*x*–(τ-180°*y*–τ)_*n*_–collect FID], using the Bruker pulse sequence (cpmgpr), where RD = 2 s, the number of loops n = 100 and the spin-echo delay τ = 400 μs, to allow spectral editing through T_2_ relaxation and therefore attenuation of broad signals. During the relaxation delay, irradiation was applied to achieve suppression of the water peak. For each spectrum, after 8 dummy scans, 128 transients were collected into 65,536 data points with a spectral width of 20 ppm and total spin–spin relaxation delay (2n.τ) of 80 ms.

### Two-dimensional NMR spectroscopic analysis

Two-dimensional (2D) NMR experiments (Claridge, 2009) were carried out for selected urine and plasma samples to aid/confirm the assignment of metabolites. The detailed parameters for the acquisition of the 2D NMR spectra are provided in Supplementary Tables 1, 2.

### NMR data processing of mouse urine and plasma

NMR spectra were processed using the software TopSpin 3.2 (Bruker Biospin, UK). Prior to applying Fourier transformation, the free induction decays (accumulated transients) were multiplied by an exponential function corresponding to a line broadening of 0.3 Hz. The 1D ^1^H NMR spectra were manually phased, baseline corrected and referenced to the chemical shift of TSP (0.0 ppm), for urine, and to the anomeric doublet of α-D-glucose at δ 5.233, for plasma samples.

The NMR data were then imported into Matlab (R2010 b, Mathworks) using in-house routines (MetaSpectra, Dr O. Cloarec, Imperial College) with a resolution of 0.00025 ppm. All subsequent data processing and analysis, unless stated otherwise, was carried out using in-house Matlab routines, written by Dr K. Veselkov's team as previously described and exemplified. (Veselkov et al., 2011, 2014) For urine samples, regions of the spectra upfield of 0.8 ppm, downfield of 10 ppm, and the spectral region containing water (δ 4.7–4.9) were omitted to eliminate the effects of background noise and variable water saturation respectively.

For the plasma NMR spectra, regions of the spectra downfield of 10 ppm and upfield of 0.8 ppm (CPMG) and 0.2 ppm (1D), as well as resonances corresponding to the water signal region (δ 4.2–5.2, 1D) and (δ 4.6–5.15, CPMG), were excluded.

All NMR spectra were normalised using “Probabilistic Quotient Normalization” (Dieterle et al., 2006) in order to compensate for differences in concentration between samples. All ^1^H NMR spectra were then aligned using recursive segment-wise peak alignment (RSPA) method. (Veselkov et al., 2009) Alignment was performed in two steps: first, a global correction was carried out using the RSPA algorithm, and second, a custom interval approach was performed for signals that exhibited high chemical shift variation, like citrate and taurine, to align peak positions in baseline-separated regions defined by the researcher. All NMR spectra were also log-transformed to convert multiplicative noise into additive noise for downstream pattern recognition analysis. (Veselkov et al., 2011).

### Mass spectrometry analysis of mouse urine samples

High-resolution ultra-performance liquid chromatography—mass spectrometry (UPLC-MS) analysis was carried out using an UPLC system coupled to an accurate-mass Quadrupole Time-of-Flight (Q-TOF) mass spectrometer. Samples were introduced into an ACQUITY UPLC HSS T3 column (2.1 mm × 100 mm, 1.8 μm, Waters, UK) with a VanGuard Pre-column (5.0 × 2.1 mm, 1.8 μm). The mobile phase consisted of 0.1% aqueous formic acid (A) and acetonitrile (ACN) and 0.1% formic acid (B). The following gradient program was used, with A + B = 100% at each timepoint: 1% B at 0–1 min, 15% B at 1–3 min, 50% B at 3–6 min, 95% B at 6–10 min, and 1% B at 10–10.1 min, followed by re-equilibration for 2 min. The flow rate was 0.5 ml/min and the injection volume was 5 μl.

Mass spectrometry was performed using a Waters Synapt G2, operating in ESI mode (positive ion) with lock mass in operation. The source temperature was set to 140°C with a cone gas flow of 90 l/h, a desolvation temperature of 350°C and a desolvation gas flow of 900 l/h. The capillary voltage was 1.50 kV for positive ionization mode and the cone voltage was 20 V. A scan time of 1 s with an inter-scan delay of 0.024 s was used for all analyses. Leucine-enkephalin at a concentration of 2 ng/μl (in 50:50 acetonitrile: 0.1% aqueous formic acid) was used as the lock-mass to ensure mass accuracy and reproducibility. The lock-spray frequency was 30 s. Mass detection was carried out in the full-scan mode with an *m/z* range from 50 to 800 in positive-ion mode. All output accurate mass measurements were then corrected for the mass of the electron, which the commercial software currently fails to do.

### Desorption electrospray ionisation mass spectrometry imaging (DESI-MSI)

Cryosections of samples were stored in closed containers at −80°C and were allowed to thaw at room temperature under nitrogen flow for 5 min prior to DESI-MS acquisition. DESI-MS analysis was performed using an Exactive Orbitrap MS (Thermo Fisher Scientific Inc., Bremen, Germany) controlled by XCalibur 2.1 software. The following instrumental parameters were used: nominal mass resolution 100,000 (mass accuracy of <4 ppm), injection time 1,000 ms, mass to charge (*m/z*) range 150-1,000, capillary temperature 250°C, capillary voltage 50 V, tube lens voltage −150 V, and skimmer voltage −40 V. DESI-MS was performed in negative- and positive-ion modes on separate adjacent tissue sections. The following DESI sprayer settings were used: sprayer to surface distance 2 mm, sprayer to MS inlet capillary distance 14 mm, solvent flow rate 1.5 μl/min, gas flow rate 7 bar, 90:10 v/v methanol/water solvent composition, electrospray potential 5 kV, and an incidence angle of 75°.

### Pattern recognition and statistical data analysis

Pattern recognition analyses were performed on the processed spectral data using Matlab (The MathWorks Inc., Natick USA). Initially, principal component analysis (PCA) of the NMR spectral data was performed to visualize group clustering, that is, overall similarities and differences between spectroscopic profiles, and to identify any abnormalities or outliers within the data set. Bi-cross validation was used to ensure that the principal components captured systematic variation not attributable to noise. (Owen and Perry, 2009) Subsequently, a supervised, multivariate analysis method, maximum margin criterion (MMC) (Veselkov et al., 2014) was employed to simultaneously maximize the variation between groups, whilst minimizing intra-group differences. “Leave-mouse-out” cross-validation with quadratic classification was used to assess the predictive capacity of the models. The spectral profiles of each animal were withheld from the dataset one at a time. The discriminating components, that is, linear combinations of metabolic features that separate classes in a mathematically optimal way, were derived based on the remaining data. The withheld profiles were then projected onto the discriminating space and assigned to the class to which they had the smallest distance via the quadratic classifier. The cross-validation was repeated until the spectral profiles of all animals were predicted. The classification accuracies (that is, confusion matrices) and predicted variance were used to assess the performance of the supervised multivariate models. Potentially discriminatory metabolites were selected using “training” profiles by one-way analysis of variance with a liberal *p*-value threshold of 0.05 (ANOVA Moyé, 2016, *p*-value < 0.05) and their collective capacity to discriminate between classes was tested on withheld (“test”) profiles using the above multivariate modelling strategy. Additionally, one-way ANOVA with a FDR of either 0.1 or 0.05, to account for multiple hypothesis testing (Benjamini, 2010), was applied to identify metabolites that individually (irrespective of other metabolites) discriminate between classes, based on suitably adjusted threshold *p*-values. Typically, the adjusted *p* values corresponding to an FDR of <10% would be significantly < 0.05.

### Metabolite identification

Metabolite identification was carried out using standard methods (Dona et al., 2016) and using information from the literature and public databases including the Chenomx NMR Suite (http://www.chenomx.com/), the Human Metabolite Database (HMDB, http://www.hmdb.ca/) (Wishart et al., 2013), the Biological Magnetic Resonance Data Bank (BMRB, http://www.bmrb.wisc.edu/metabolomics/) (Ulrich et al., 2008), the Birmingham Metabolite Library (BML, http://www.bml-nmr.org/) (Ludwig et al., 2012), and COLMAR (Complex Mixture Analysis by NMR, http://spin.ccic.ohio-state.edu/index.php/colmarm/index) (Bingol et al., 2015). A series of 2D NMR experiments, including J-resolved, correlation spectroscopy (COSY), TOCSY, HSQC, and HMBC (Claridge, 2009), were acquired for a number of samples in order to assist or confirm the identification of a range of metabolites. The identities of the key discriminating metabolites were also confirmed by the spiking of authentic standards into urine and plasma samples. Confidence in the identification of these known metabolites was assessed using the new metabolite identification carbon efficiency (MICE) (Everett, 2015) and topological MICE (tMICE) (Sanchon-Lopez and Everett, 2016) methods.

### Identification of 6-hydroxy-6-methyl-heptan-3-one (6H6MH3O)

One of the most distinctive, ageing-correlated features of the low-frequency region of the ^1^H NMR spectra of male WT and FMO5 KO mice was a sharp singlet at ca. 1.209 ppm. Extensive 1D and 2D NMR analyses, confirmed by orthogonal high-resolution UPLC-MS analyses proved that this signal and others were due to 6H6MH3O. This metabolite has previously only been identified via its degradation products in the headspace above and, after derivatisation, within male urine samples, by GC-MS, but has not previously been identified by NMR spectroscopy and is not present in the BML, BMRB, or HMDB databases. This metabolite is unusual in that it exists in two distinct tautomeric forms.

**Figure:**
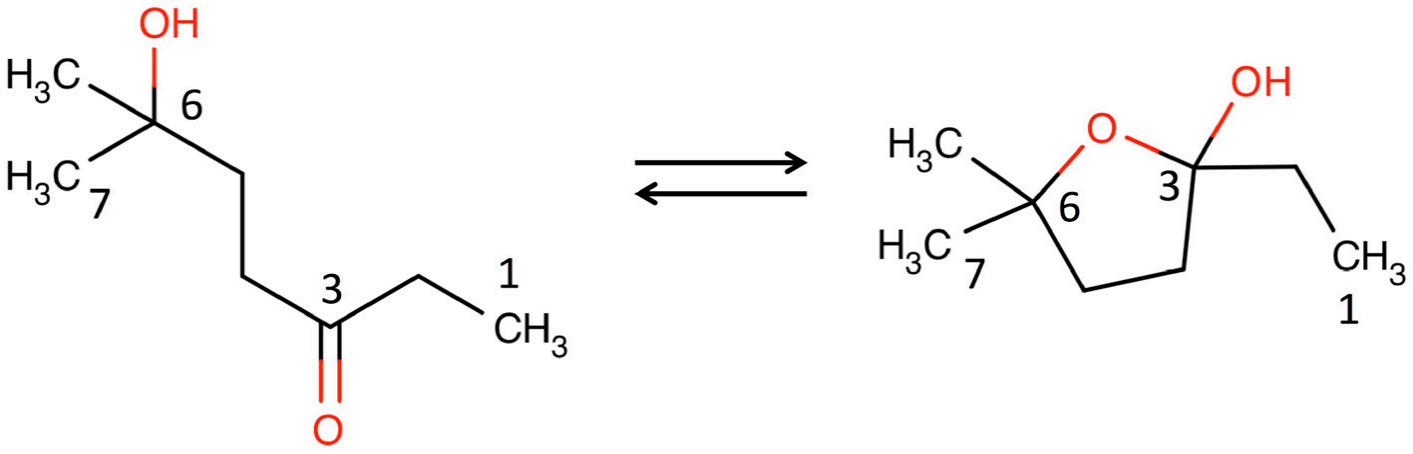
6H6MH3O shown in the linear, achiral, hydroxy-ketone tautomeric form (left), and the cyclic, chiral, hemi-ketal tautomeric form (right).

Across the two tautomers, a total of 24 bits of metabolite identification information were obtained. Thus, in this 8-carbon metabolite, the MICE (Everett, 2015) value is 24/8 = 3.0; and 6H6MH3O is, thus, confidently identified. A topological MICE analysis (Sanchon-Lopez and Everett, 2016) of the metabolite identification is not reported, as the two tautomers each have different molecular topologies.

The metabolite is not commercially available, so an authentic sample of the metabolite (Tashiro et al., 2008) was obtained from Professor Kenji Mori, Emeritus Professor, University of Tokyo, and it was also independently synthesized by Enamine, Ukraine. The NMR data for the authentic metabolite match those for the metabolite found in the mouse urine with high precision (Supplementary Table 3). For the nine ^13^C and eight ^1^H NMR shifts reported across the two tautomers, the average differences and the standard deviations of those differences were 0.156 ± 0.106 and 0.002 ± 0.001 ppm respectively, well within expected shift deviations for the same metabolite in different matrices: buffered urine compared with buffer (Dona et al., 2016; Sanchon-Lopez and Everett, 2016).

The 6H6MH3O metabolite is, thus, unambiguously identified (MSI identification level 1) (Sumner et al., 2007). Full information on the identification of both tautomers of this ageing biomarker is given in the Supplementary Information.

## Results

### Metabolic signature of ageing in the urinary metabolome

**Figure 1:**
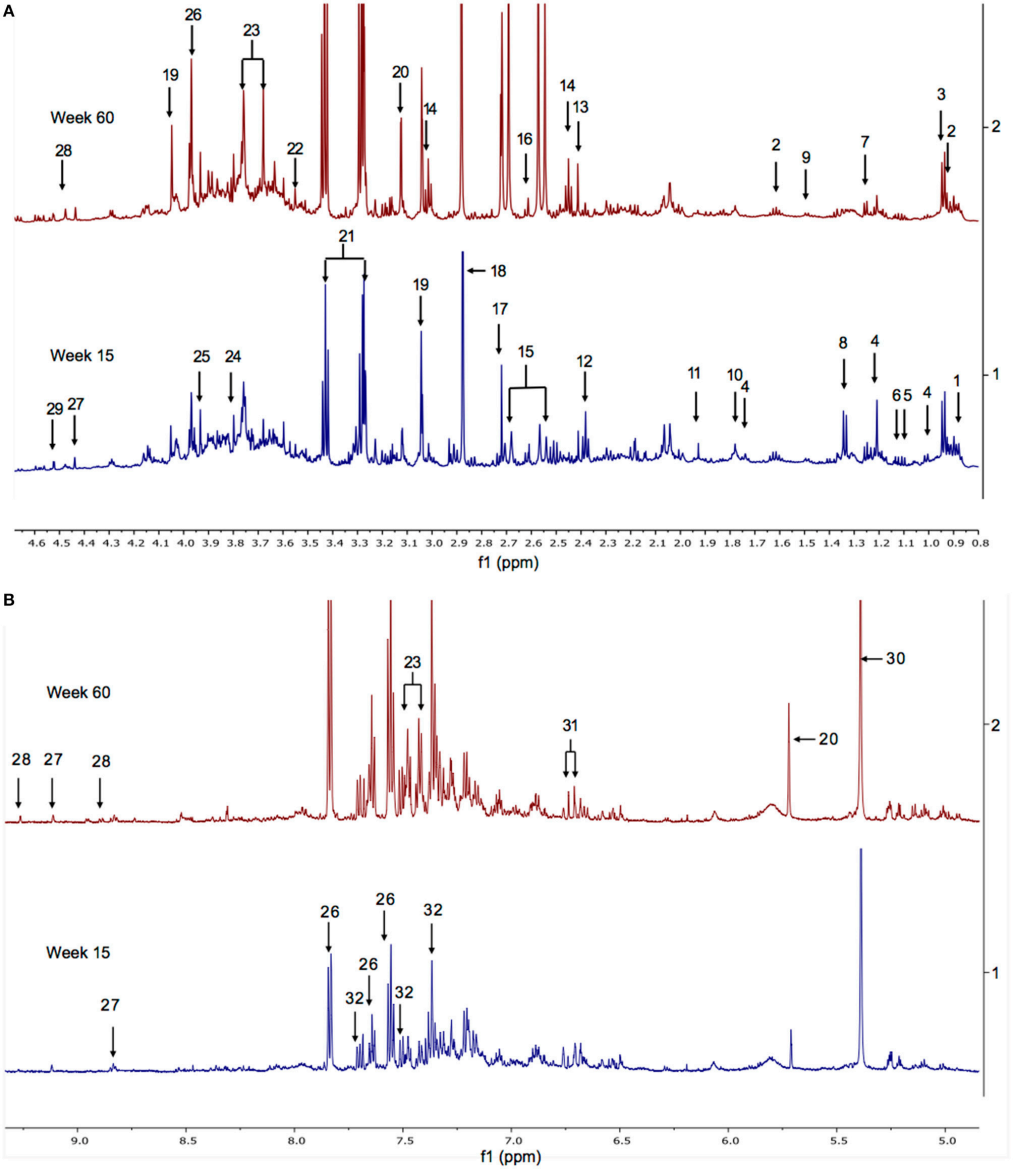
**(A)** The aliphatic region of ^1^H NMR spectra of urine from representative WT mice at weeks 15 (bottom) and 60 (top). Key: 1. hexanoylglycine; 2. *N*-butyrylglycine; 3. *N*-isovalerylglycine; 4. 6-hydroxy-6-methyl-heptan-3-one; 5. 3-methyl-2-oxovalerate; 6. 2-oxoisovalerate; 7. fucose; 8. lactate; 9. alanine; 10. putrescine; 11. acetate; 12. ureidopropionate; 13. succinate; 14. 2-oxoglutarate; 15. citrate; 16. methylamine; 17. dimethylamine; 18. trimethylamine; 19. creatinine; 20. cis-aconitate; 21. taurine; 22. glycine; 23. phenylacetylglycine; 24. guanidoacetate; 25. creatine; 26. hippurate; 27. trigonelline; 28. 1-methylnicotinamide (1MNA); 29. ascorbate. Supplementary Figure 19 shows the corresponding spectra from all of the mice in the weeks 15 and 60 cohorts. **(B)** The aromatic region of the ^1^H NMR spectra of urine from male WT mice at weeks 15 (bottom) and 60 (top). Key: 20. cis-aconitate; 23. phenylacetylglycine; 26. hippurate; 27. trigonelline; 28. 1-methylnicotinamide (1MNA); 30. allantoin; 31. cinnamoylglycine; 32. 3-indoxylsulphate. Supplementary Figure 20 shows the corresponding spectra from all of the mice in the weeks 15 and 60 cohorts.

The ^1^H NMR spectra of urine samples from WT and FMO5 KO mice contain hundreds of resonances from dozens of metabolites, of which ca. 100 have been confidently identified (Everett, 2015; Sanchon-Lopez and Everett, 2016). Typical spectra of the urine from WT mice at 15 and 60 weeks of age are shown in Figure 1.

**Figure 2:**
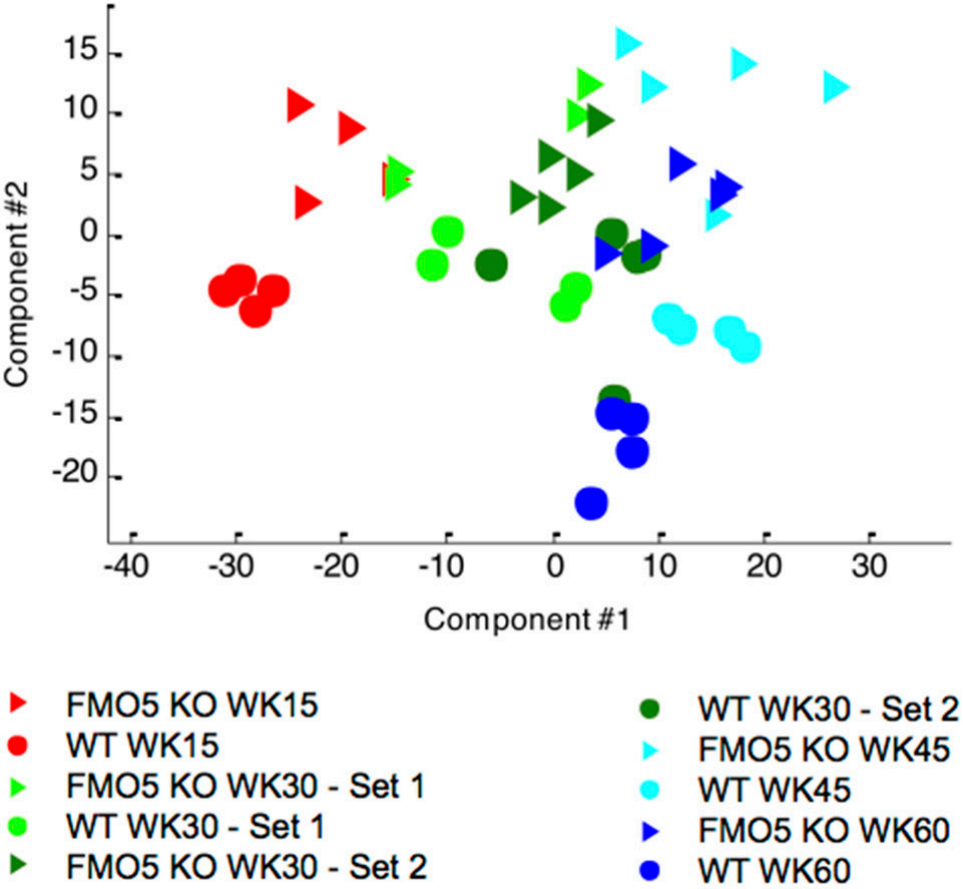
A PCA scores trajectory plot of the 600 MHz ^1^H NMR spectra of urine from male WT and FMO5 KO mice at different time points. PC1 explains 32.2 and PC2 11.6% of the variance.

To visualize the effect of ageing on mouse urinary metabolite profiles, the NMR data were analysed using an unsupervised statistical technique, principal components analysis (PCA). This analysis showed distinct, time-dependent metabolic changes along principal component 1 (PC1) from week 15 to week 45, and then an excursion along PC2 from week 45 to 60, although the latter much less so for the FMO5 KO mice. There is tendency to a higher degree of inter-individual variation within the FMO5 KO relative to the WT mice at most time points and a clear separation between FMO5 KO and WT mice, mainly along PC2 at all time points (Figure 2).

To determine ageing-related metabolites in urine, ANOVA with *p*-values adjusted for a FDR of 0.1, that is, 10%, was performed on NMR spectra acquired at every two adjacent time points, as well as between the first and the last time points with a more stringent and conservative FDR of 0.05, that is, 5%, for both KO and WT mice, to identify metabolites that individually (irrespective of other metabolites) discriminate between classes (Table 1). We also determined potentially discriminatory metabolites by one-way analysis of variance without an FDR filter, simply using a more liberal *p*-value threshold of 0.05 (ANOVA, *p*-value < 0.05) and their collective capacity to discriminate between classes was tested using the above multivariate modelling strategy. These *potentially* discriminating metabolites are indicated with the letter p in Table 1.

**Table 1:** Statistically significant (ANOVA) metabolite changes in pair-wise comparison of adjacent time points as well as between the first and the last time points in urine samples from male FMO5 KO and WT mice.

**Compounds**	**δ^1^H (multiplicity) in ANOVA**	**WT 30 vs. 15**	**KO 30 vs. 15**	**WT 45 vs. 30**	**KO 45 vs. 30**	**WT 60 vs. 45**	**KO 60 vs. 45**	**WT 60 vs. 15**	**KO 60 vs. 15**
**ORGANIC ACIDS**									
Acetate	1.92 (s)	↓	–	–	–	–	–	↓	↓
Ascorbate	4.52 (dd)	↑	↑	↓	–	–	–	↑p	–
Isovalerate	0.92 (d)	↓	↓	–	–	–	–	↓	↓
Lactate	1.34 (d)	–	–	–	–	–	–	↓	↓
3-methyl-2-oxovalerate	1.10 (d)	–	↓	–	–	–	–	–	↓
**ACYL-GLYCINE CONJUGATES**									
Butyrylglycine	0.93 (t), 1.62 (m)	–	↓	–	–	–	–	↓	↓
Cinnamoylglycine	6.73 (d)	↑	–	–	–	–	–	–	–
Hexanoylglycine	0.88 (t)	–	↓	–	–	–	–	↓	↓
**ALCOHOLS, SUGARS, AND KETONES**									
6-hydroxy-6-methylheptan-3-one (6H6MH3O)	1.01 (t), 1.21(s), 1.74 (m)	↓	↓p	↓p	↓	–	↓p	↓	↓
Arabinose	4.53 (d)	–	–	–	–	↑	–	↑	–
D-xylose	4.59 (d), 5.21 (d)	–	–	–	–	↑	↑p	↑	↑
D-glucose	4.66 (d)	–	–	–	–	↑	↑	↑	↑
D-glucuronate	4.65 (d)	–	–	–	–	↑	↑	↑	↑
Fucose	1.25(d)	↑	–	↓	–	–	–	–	–
**CITRATE CYCLE INTERMEDIATES**									
2-oxoglutarate	2.45 (t), 3.01(t)	↑p	↑p	–	–	–	–	↑	↑
Citrate	2.56 (d), 2.70 (d)	↑p	↑	↑	–	–	–	↑	↑
Succinate	2.41(s)	↑p	↑p	–	–	–	–	↑	↑
**AMINES, AMIDES, AMINO ACIDS, AND RELATED**									
1-methyl nicotinamide (1MNA)	4.47 (dd)	–	–	–	–	↑	–	↑	↑
Allantoin	5.4 (s)	–	–	–	–	–	–	↑	↑
Dimethylamine	2.73 (s)	↑p	–	–	–	–	–	↑	↑
Creatinine	3.04 (s), 4.05 (s)	↑	↑	–	–	–	–	↑	↑
Putrescine	1.78 (m)	–	↓	↑p	–	↓	–	–	↓
Taurine	3.27 (t), 3.43 (t)	↑	–	↑	–	–	–	↑	–
Trimethylamine	2.88 (s)	↑	↑p	–	–	–	–	–	↑
Trimethylamine-*N*-oxide	3.27 (s)	–	–	–	–	–	–	↑	↑
Trigonelline	4.44 (dd)	–	–	–	–	↑	↑p	↑	↑
Ureidopropionate	2.38 (t), 3.31 (t)	↓	–	–	–	–	–	↓	–
**MAMMALIAN MICROBIOME CO-METABOLITES**									
Hippurate	7.56,7.64, 7.84	–	↑	–	–	–	–	↑	↑
Indoxylsulphate	7.51, 7.71	↑	–	–	–	–	↓p	↑	–
4-cresol glucuronide	2.30 (s), 5.08 (d), 7.0	↑	–	–	–	↓	↓p	–	–
4-cresol sulphate	2.35 (s),	↑	–	–	–	↓	↓p	–	–
Phenylacetylglycine	7.37 (m), 7.43 (dd)	↑	–	–	–	–	–	↑	↑
**UNKNOWNS**									
U1	1.228 (d)	–	–	↓	–	–	–	–	–
U2	1.31(m)	–	↓	–	–	–	–	–	–
U3	1.83 (m)	–	–	↑	–	–	–	–	–
U4	2.064 (s)	↓	–	–	–	–	–	–	–
U5	2.182 (s)	–	–	–	–	–	–	↓	–
U6	2.74 (s)	↓	–	–	–	–	–	–	–
U7	2.78 (s)	–	–	↑	–	–	–	–	–
U8	4.41 (d)	–	–	–	–	–	–	–	↑
U9	4.575 (d)	–	–	–	–	–	↑	↑	–
U10	4.99 (d)	–	–	–	–	–	–	↑	–
U11	5.084 (d)	–	–	–	–	–	–	↑	–
U12	5.09 (d)	–	–	–	–	–	–	↑	–
U13	8.06 (d)	–	–	–	–	–	–	–	↑

*(↑) Indicates an increase in the concentration of metabolites, and a (↓) a decrease in the concentration of metabolites and (–) indicates no significant difference. Arrows (↑↓) indicate statistically significantly discriminating metabolites with p-values adjusted for a false discovery rate (FDR) of 0.1 generally and a more stringent FDR of 0.05 for the comparisons of weeks 60 and 15 (last two columns). The “p-annotated” arrows (↑p, ↓p) indicate potentially discriminating metabolites with a simple, unadjusted p-value threshold of 0.05. Full NMR data for all of the identified discriminating metabolites is given in Supplementary Table 4*.

The urinary metabolome of 30-week-old WT mice showed statistically significantly (FDR < 10%) higher concentrations of taurine, fucose, creatinine, ascorbate, and mammalian microbiome co-metabolites, including phenylacetylglycine, 4-cresol glucuronide, 4-cresol sulphate, indoxylsulphate, cinnamoylglycine, and trimethylamine, along with statistically significantly (FDR < 10%) lower concentrations of 6H6MH3O, acetate, isovalerate, ureidopropionate, *N*-acetyl protein at 2.065, and other unknown signals at 2.74 ppm, when compared with WT mice at week 15. The other metabolites that were found to be potentially discriminating with a more liberal, unadjusted *p*-value threshold of 0.05 were citrate, succinate, 2-oxoglutarate and dimethylamine, all of which increased as the WT mice aged from 15 to 30 weeks (Supplementary Figure 6).

The urinary metabolome of 30-week-old FMO5 KO mice showed statistically significantly (FDR < 10%) elevated concentrations of citrate, creatinine, hippurate, ascorbate and, potentially, succinate, 2-oxoglutarate and trimethylamine, along with statistically significantly (FDR < 10%) lower concentrations of putrescine, hexanoylglycine, isovalerate, butyrylglycine, 3-methyl-2-oxovalerate and an unknown peak at 1.31 ppm and, potentially, 6H6MH3O, when compared with the urinary profile of FMO5 KO mice at week 15 (Supplementary Figure 7).

The metabolic composition of the urine was more stable as mice aged from 30 to 45 weeks and only elevated concentrations of taurine and citrate, and of the unknowns U3 and U7, and lower concentrations of fucose, ascorbate and of the unknown U1 were significantly different in WT mice at week 45 compared with WT mice at week 30 (Supplementary Figure 8), whereas higher concentrations of putrescine and lower concentrations of 6H6MH3O were observed at week 45 with a more liberal, unadjusted *p*-value threshold of 0.05.

In FMO5 KO mice, only lower concentrations of 6H6MH3O were found to be statistically significantly different (FDR < 10%), as mice aged from 30 to 45 weeks (Supplementary Figure 9).

At 60 weeks, WT mice exhibited statistically significantly (FDR < 10%) higher concentrations of D-xylose, D-glucose, D-glucuronate, arabinose, 1-methylnicotinamide (1MNA) and trigonelline and statistically significantly (FDR < 10%) lower concentrations of putrescine, 4-cresol sulphate and 4-cresol glucuronide, than at week 45 (Supplementary Figure 10).

Correspondingly, FMO5 KO mice at week 60 showed statistically significantly (FDR < 10%) higher concentrations of D-glucose, D-glucuronate, and U9 (an unknown peak at 4.57), along with potentially higher concentrations of trigonelline and xylose and potentially lower concentrations of indoxylsulphate, 4-cresol glucuronide, 4-cresol sulphate and 6H6MH3O, relative to 45-week-old mice (*p* < 0.05, but not adjusted for FDR, Supplementary Figure 11).

**Figure 3:**
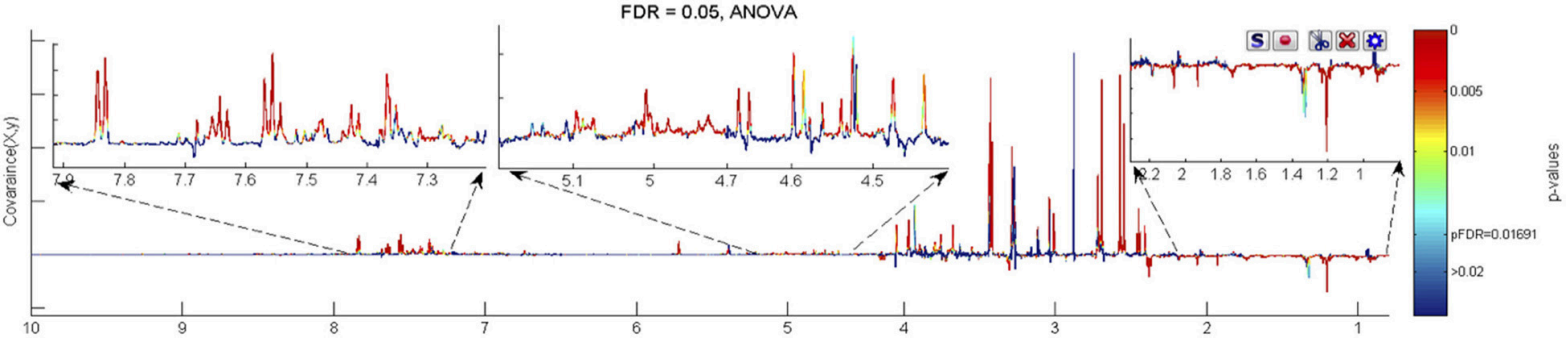
The ANOVA plot for the 600 MHz urine ^1^H NMR spectra of male WT mice at week 60 age vs. the corresponding spectra of WT mice at week 15 age, showing positive peaks for those metabolite signals that are more intense at week 60 than at week 15, and negative peaks for those metabolite signals that are less intense. The signals are colour coded by the *p*-value from the ANOVA analysis. In this case a false discovery rate cut off of 5% was used and the threshold *p*-value for significant difference was calculated as 0.0169, corresponding to those signals with colouring to the “red side” of light blue.

**Figure 4:**
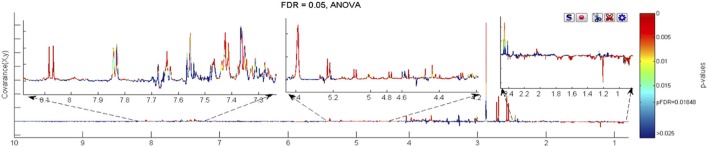
The ANOVA plot for the 600 MHz urine ^1^H NMR spectra of male FMO5 KO mice at week 60 age vs. the corresponding spectra of WT mice at week 15 age, showing positive peaks for those metabolite signals that are more intense at week 60 than at week 15, and negative peaks for those metabolite signals that are less intense. The signals are colour coded by the *p*-value from the ANOVA analysis. In this case a false discovery rate cut-off of 5% was used and the threshold *p*-value for significant difference was calculated as 0.0185, corresponding to those signals with colouring to the “red side” of light blue.

The most profound metabolic differences were observed in models constructed to analyse the overall changes between weeks 15 and 60. The dominant ageing-related changes observed in *both* FMO5 KO and WT mice were statistically significant (FDR 5%) increases in creatinine, dimethylamine, 1MNA, allantoin, D-xylose, D-glucose, D-glucuronate, tricarboxylic acid intermediates such as citrate, 2-oxoglutarate and succinate, and host–microbiota metabolites including hippurate, phenylacetylglycine, trigonelline and trimethylamine *N*-oxide (TMAO), along with statistically significant decreases in lactate, acetate, isovalerate, hexanoylglycine, butyrylglycine, butanone, and 6H6MH3O (Figures 3 and 4).

Unique, statistically significant, ageing-related changes in the urines of WT mice from weeks 15 to 60 were: elevated concentrations of taurine, indoxylsulphate, arabinose, and unknown peaks U9 to U12, as well as reduced concentrations of ureidopropionate and an unknown peak U5 (Figure 3).

Unique, statistically significant, ageing-related changes in the urines of FMO5 KO mice only from weeks 15 to 60 were: higher concentrations of trimethylamine and unknown peaks U8 and U13, and lower concentrations of α-3-methyl-2-oxovalerate and putrescine (Figure 4).

The signals from 6H6MH3O were relatively broad, that is, large half band width, compared with those of other small metabolites, as would be expected for a sex pheromone in fast exchange with the major urinary proteins (MUPs) present at relatively high concentrations in male mouse urine. In agreement with this, ultrafiltration of three male WT and FMO5 KO urines showed a half band-width reduction from 1.94 ± 0.06 Hz to 1.36 ± 0.06 Hz (*p* = 0.0008) and from 1.18 ± 0.05 Hz to 0.90 ± 0.05 Hz (*p* = 0.039) for the methyl signals of the linear, achiral, acyclic tautomer at ca. 1.208 and 1.017 ppm respectively. These statistically significant reductions in signal half band width for 6H6MH3O contrasted with the lack of statistically significant half band width changes for the singlet signal for trimethylamine at 2.88 ppm (0.73 ± 0.02 Hz unfiltered vs. 0.66 ± 0.05 Hz filtered, *p* = 0.11) and the central line of the triplet for taurine at 3.43 ppm (0.73 ± 0.02 Hz unfiltered vs. 0.66 ± 0.05 Hz filtered, *p* = 0.61), indicating a lack of significant protein binding to urinary proteins for trimethylamine or taurine.

### Metabolic signature of ageing in the plasma metabolome

**Figure 5:**
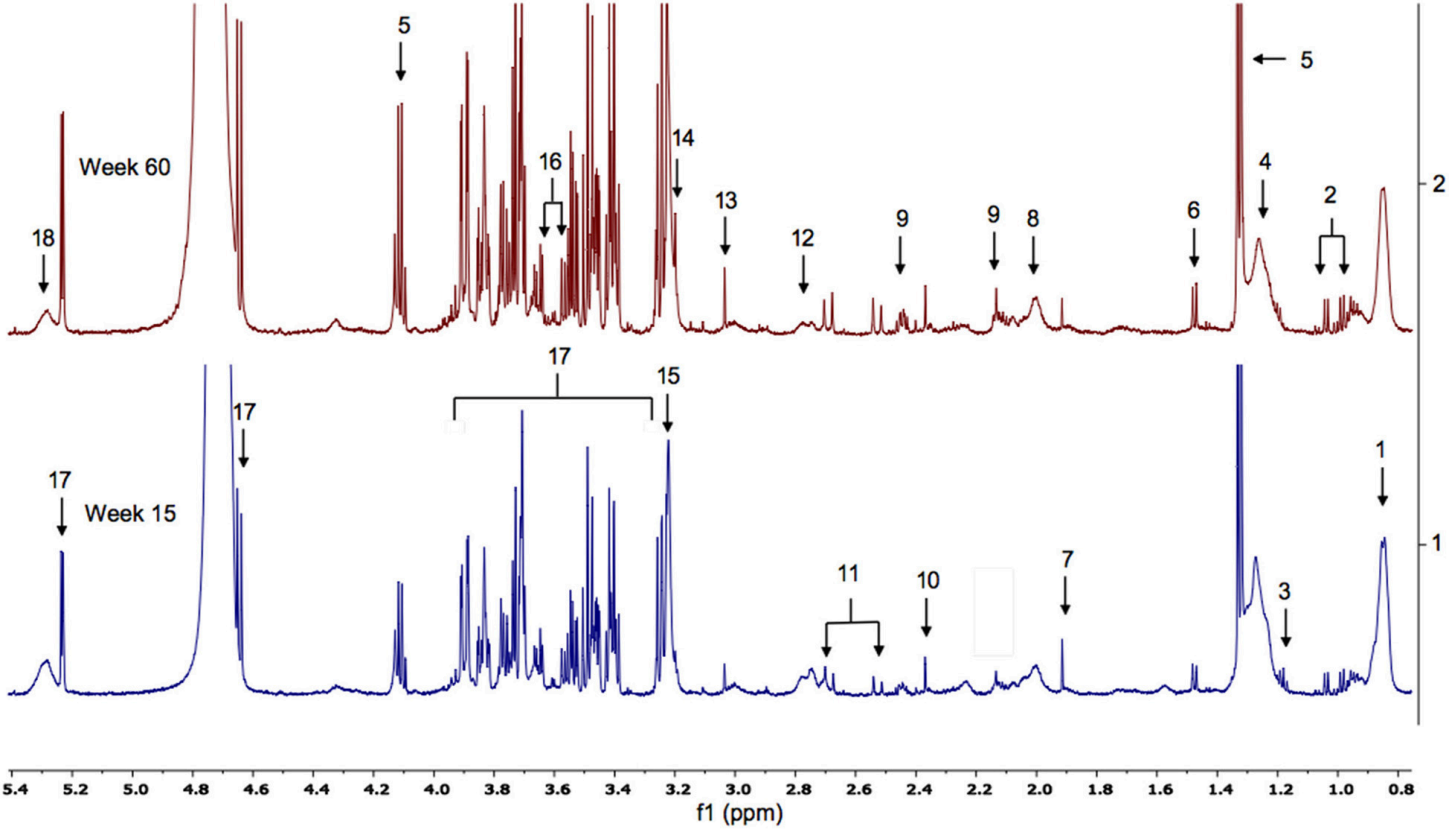
Typical 600 MHz (1D-CPMG) ^1^H NMR spectra of plasma (aliphatic region) from male, wild-type mice at weeks 15 and 60. Key. 1. LDL/VLDL; 2. valine; 3 ethanol; 4. LDL/VLDL; 5. lactate; 6. alanine; 7. acetate; 8. lipid; 9. glutamine; 10. pyruvate; 11.citrate; 12. lipid; 13. creatine; 14. choline; 15. phosphocholine-containing molecule; 16. glycerol; 17. glucose.

**Figure 6:**
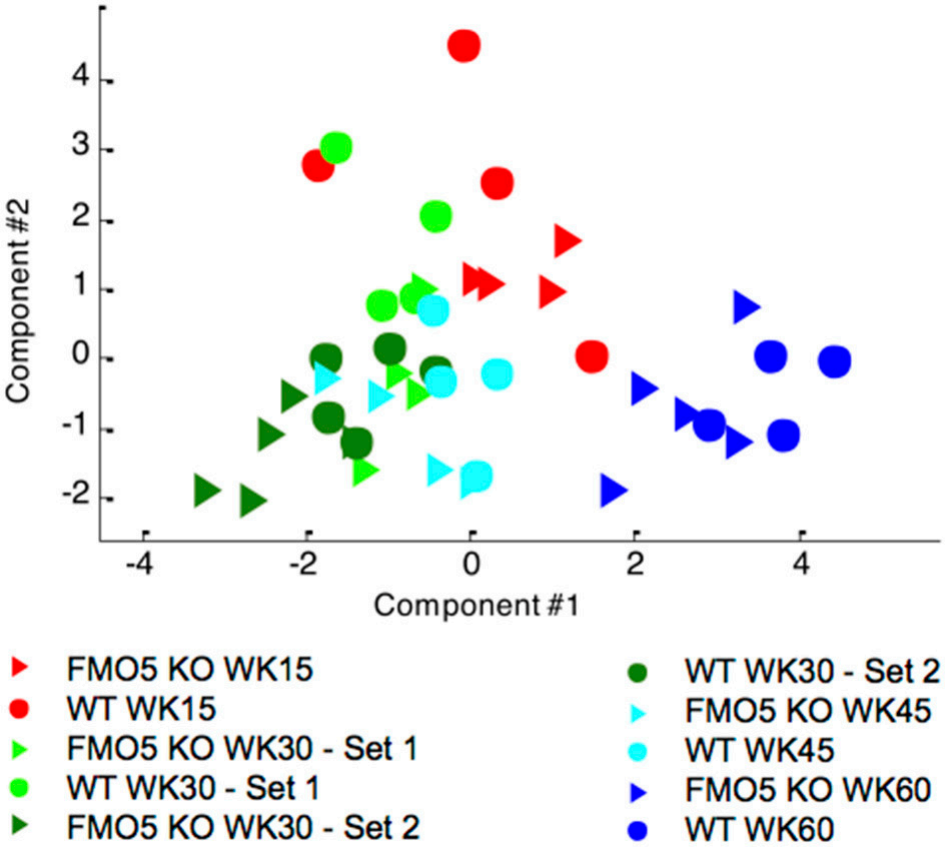
Unsupervised PCA scores trajectory plot of the 600 MHz plasma ^1^H NMR spectra from male FMO5 KO and WT mice at different time points. PC1 explains 37.9% of the variance, PC2 explains 22.5%.

Typical ^1^H NMR spectra of plasma samples from WT mice at weeks 15 and 60 are shown in Figure 5. As was the case for urine, the plasma NMR data were analysed using PCA, an unsupervised and unbiased statistical technique. Figure 6 shows a PCA scores trajectory plot for the plasma from both WT and FMO5 KO mice. Clear, age-related metabolic changes were observed for both FMO5 KO and WT mice. In contrast to the urine results (Figure 2), the plasma PCA trajectory plot shows some more variance in the WT mice plasma at week 15 relative to the corresponding FMO5 KO plasma but, after that, less difference in within-group variances at each time point. The metabolic trajectory moves “south-west” from week 15 to week 30 and then “east” i.e., left to right, across PC1 to weeks 45 and 60.

As in urine, discriminatory metabolites associated with ageing in mouse plasma were determined for every two adjacent time points, as well as between the first and the last time points, for both FMO5 KO and WT mice, by one-way ANOVA with an FDR of 10%. Potentially discriminatory metabolites (denoted by the letter p associated with the arrows in Table 2) were also determined by ANOVA without an FDR filter and with simply a more liberal *p*-value threshold of 0.05.

**Table 2:** Metabolic changes in pair-wise comparison of adjacent time points as well as between the first and the last time points in plasma samples from male FMO5 KO and WT mice.

**Compounds**	**Chemical shifts in ppm & (multiplicity)**	**WT**	**KO**	**WT**	**KO**
		**week 60 vs. 45**		**week 60 vs. 15**	
**AMINES, AMIDES, AMINO ACIDS, AND RELATED**					
Isoleucine	0.94 (t), 1.01 (d), 1.98 (m)	–	–	↑	↑
Leucine	0.95 (d), 0.97 (d), 1.73 (m)	↑p	↑	↑	↑
Valine	0.99 (d), 1.04 (d), 3.60 (d)	↑p	↑	↑	↑
Alanine	1.47 (d), 3.78 (q)	–	↑	–	–
Creatine	3.04 (s)	–	↑	↑	–
Choline	3.20 (s)	↑	↑	↑p	↑
Glutamine	2.45 (m), 2.13 (m)	–	↑	↑	↑
Trimethylamine	2.89 (s)	–	↑	–	–
**LIPIDS**					
Lipid, mainly VLDL, (CH_2_CH_2_CO)	1.58	–	–	↓	–
Lipid (CH_2_C = C)	2.0	–	↑	–	↑
Lipid (CH_2_CO)	2.23	–	–	↓	–
Lipid (C = CCH_2_C = C)	2.74	↓	↓	↓	↓
Unsaturated lipid	5.28	↓	↓	–	↓
GPC or PtdCho	3.22 (s), 3.62	↑	↑	↑p	↑
Glycerol	3.56 (dd), 3.65 (dd)	↑	↑	↑p	↑
**ORGANIC ACIDS**					
3-hydroxyisobutyrate	1.07 (d), 2.47 (m)	–	↑	–	–
Acetate	1.91 (s)	–	↑	–	–
Lactate	1.32 (d), 4.10 (q)	–	↑	↑	↑
**TCA INTERMEDIATES**					
Citrate	2.53 (d), 2.69 (d)	–	↑	↑p	↑
Succinate	2.41 (s)	–	–	↑	↑
**ALCOHOLS**					
Ethanol	1.18 (t), 3.65 (q)	↓	–	↓	↓
**UNKNOWN**					
U1 *N*–acetyl glycoprotein derivative	2.08 (m)	–	↑	–	–
U2	2.13 (s)	↑	↑	↑	–
U3	3.11 (s)	–	↑	–	↑

*(↑) Indicates an increase and (↓) a decrease in the concentration of metabolites, with p-value adjusted for FDR of 0.1, and ↑p, ↓p indicates potentially discriminating metabolites based solely on the p-value threshold of 0.05. (–) Indicates no significant difference metabolite concentration. Full NMR data for all of the identified discriminating metabolites is given in Supplementary Table 5. No columns are included for WT or FMO5 KO mice for weeks 30 compared with 15 or week 45 compared with week 30, as there were no significant metabolite concentration changes*.

For both WT and FMO5 KO mice, no age-related discriminating metabolites were observed up to 45 weeks (see Supplementary Figures 12–15). However, significant plasma metabolic changes were observed as mice aged from 45 to 60 weeks of age.

WT mice at week 60 showed statistically significantly (FDR 0.1) elevated concentrations of choline, choline-containing metabolites including glycerophosphocholine (GPC) and/or phosphatidylcholine (PtdCho), glycerol and an unknown U2, and potentially increased (*p* < 0.05, unadjusted for FDR) concentrations of the branched-chain amino acids (BCAA) leucine, and valine, along with statistically significantly decreased concentrations of ethanol and unsaturated lipid at 2.74 and 5.28 compared with week 45 (Supplementary Figure 16).

FMO5 KO mice at week 60 showed statistically significantly (FDR 0.1) elevated concentrations of 3-hydroxyisobutyrate, acetate, lactate, citrate, glutamine, alanine, choline, trimethylamine (TMA), creatine, BCAAs including leucine and valine, choline-containing metabolites including glycerophosphocholine (GPC) and/or phosphatidylcholine (PtdCho), glycerol, unsaturated lipids at 2.0 ppm (CH_2_C = C), along with unknown metabolites at 2.08 [U1, *N*-acetyl glycoprotein (NAG)-associated resonances], U2 and U3. The week 60 FMO5 KO mice also exhibited statistically significantly (FDR 0.1) reduced concentrations of unsaturated lipid at 2.74 ppm (C = CCH_2_C = C) and 5.28, compared with week 45 (Supplementary Figure 17).

A model was also constructed between 60- and 15-week-old mice. Both FMO5 KO and WT mice showed higher plasma concentrations of lactate, choline (WT potentially discriminating), glutamine, choline-containing metabolites, including GPC and/or PtdCho (WT potentially discriminating), glycerol (WT potentially discriminating), the BCAAs isoleucine, leucine and valine, the citrate cycle intermediates citrate (WT potentially discriminating) and succinate, along with lower concentrations of ethanol, at week 60 relative to week 15. WT mice alone also showed elevated concentrations of creatine and an unknown metabolite U2, along with lower concentrations of lipid at 1.58 (mainly VLDL, CH_2_CH_2_CO) and 2.23 ppm (CH_2_CO, Supplementary Figure 21), whereas FMO5 KO mice alone exhibited elevated concentrations of lipid at 2.0 ppm (CH_2_C = C), and decreased concentrations of unsaturated lipid at 5.28, at week 60 vs. week 15 (Supplementary Figure 22).

## Discussion

An NMR-based metabonomics approach was applied to simultaneously study age-related differences in urinary and plasma metabolic profiles of both male FMO5 KO and male WT C57BL/6J mice, in order to identify a metabolic signature of ageing and to determine differences in metabolic ageing between the two genotypes. The global metabonomics overview of the urine and plasma showed clear age-related changes in the metabolic composition of both biofluids and differences between the changes observed in the WT and FMO5 KO mice, the latter having been found to exhibit slowed metabolic ageing (Gonzalez Malagon et al., 2015). For both plasma and urine samples, and for both FMO5 KO and WT mice, the most profound age-related metabolic differences were observed for models constructed between mice at 15- and 60-weeks old.

### Ageing-associated changes in urinary metabolite profiles

In urine, there were more numerous, statistically significant (FDR 0.1) metabolite changes for early samples (week 30 vs. week 15) relative to later samples (week 45 vs. week 30 especially and week 60 vs. week 45). The early differences possibly reflect changes corresponding to the development of the mice from young adults to middle age, but by week 30 the composition of the urine was more stabilised and fewer significant metabolic differences were observed as a function of ageing to week 45, particularly in the case of FMO5 KO animals. However, for all time-point comparisons, there were always more statistically significant changes in the urines of the WT relative to the FMO5 KO mice. This observation is in agreement with the phenotype of FMO5 KO animals in which the effects of disruption of the *Fmo5* gene were shown to reduce metabolic ageing (Gonzalez Malagon et al., 2015). The global PCA (Figure 2) also shows that for both WT and FMO5 KO mice, the urinary metabolic trajectory across PC1 stops by week 45 and from week 45 to week 60, the metabolic trajectory is along PC2, but to a much lesser extent for the FMO5 KO mice.

In urine, metabolic signatures of ageing were characterized by alterations in the concentrations of the sex pheromone 6-hydroxy-6-methyl-heptan-3-one (6H6MH3O), metabolites associated with the citric acid cycle, and with fatty-acid, amino-acid and nucleotide metabolism, including changes to allantoin, ureidopropionate, and 1-methylnicotinamide and carbohydrate-related metabolites, as well as mammalian–microbiome co-metabolites.

Based on multivariate statistical analysis, 6H6MH3O was found to be one of the most important metabolites whose concentration reduced significantly with ageing. It is also one of the most abundant metabolites observable in the high-frequency region of the ^1^H NMR spectra of young, male WT or FMO5 KO mice. It is thus surprising that this metabolite is absent from all of the major metabolite databases, and to the best of our knowledge has not been reported previously in NMR-based studies of C57BL/6 mice. Except for WT mice at week 60 vs. week 45, this metabolite was found to be decreased, either potentially (*p* < 0.05) or statistically significantly (with additional, more stringent FDR < 10%) in both FMO5 KO and WT mice as they aged, at each stage examined (see Table 1). This observation is in agreement with Osada et al. (2008) who reported that urinary concentrations of 6H6MH3O in male C57BL/6J mice dropped significantly as they aged from 3 to 8, then up to 28 months, as measured by headspace GC-MS analysis of 6H6MH3O degradation products. Somewhat surprisingly, Schaeffer et al reported by GC-MS-based headspace analysis that a tentatively identified dihydrofuran degradation product of 6H6MH3O was significantly increased in concentration at 8 weeks relative to 4 weeks of age in C57BL/6J-H-2^b^ mice (Schaefer et al., 2010), but this was at an early age.

6H6MH3O is one of a number of volatile pheromones (Liberles, 2014) that bind to male mouse MUPs (Phelan et al., 2014), and are known to be involved in social and sexual communication and control. The binding constant of 6H6MH3O to a variety of mouse MUPs is relatively weak and in the range ca. 50 to ca. 200 uM (Sharrow et al., 2002). The mode of binding of this pheromone to MUP1 has also been elucidated by high-resolution X-ray crystallography and clearly shows the binding is specifically to the linear, achiral, hydroxy-ketone tautomer (see Materials and Methods; Timm et al., 2001). In agreement with this finding, ultrafiltration of male WT and FMO5 KO urine showed statistically significant decreases in the half band width of the singlet signal for the gem-dimethyl groups and the broad triplet for the ethyl methyl group in the linear, hydroxy-ketone tautomer, but no significant reductions in half band width for the signals of trimethylamine and taurine, which were used as controls. Ultrafiltration removes significant amounts of MUPs from the mouse urine. As a consequence of this, metabolites such as 6H6MH3O, that are in fast exchange with the MUPs, when present, will move to an environment where they are in free solution, with no contribution to their motional characteristics from binding to the MUPs. The metabolite will thus have, on average, a significantly reduced molecular correlation time and sharper signals with reduced half band width. 6H6MH3O has also been identified in mouse body odour as well as urine (Röck et al., 2006).

We believe that ours is the first direct biofluid identification of this important, high-abundance metabolite, and of *both* of its tautomers. The metabolite was previously identified by extraction and derivatization, or by headspace sampling, both methods followed by GC-MS analysis, which suffers from the issue of significant metabolite degradation, due to the thermal instability of 6H6MH3O to dehydration on high-temperature GC columns (Harvey et al., 1989; Novotny et al., 1999). Finally, through ultrafiltration, we have demonstrated that the linear, achiral tautomer of 6-hydroxy-6-methylheptan-3-one is in fast exchange with large macromolecules that we assume are MUPs in urine solution, through the reduction in signal half band width on ultrafiltration of the urine to remove the MUPs and any other biological macromolecules.

In addition, it is known that different strains of mice exhibit different arrays of pheromones and MUPs (Kwak et al., 2012), with BALB/b mice being reported to have lower concentrations of urinary 6H6MH3O relative to C57BL/6J mice. In agreement with this finding, the NMR spectra from a recent ageing study on BALB/c mice showed an absence of any significant singlet signal at ca. 1.21 ppm (that would correspond to the *gem*-dimethyl groups of the hydroxyl-ketone tautomer of 6H6MH3O) and the compound was not reported as being significantly associated with ageing from 3 to 16 months in these mice (Calvani et al., 2014). Furthermore, in addition to being present naturally at lower concentrations in male BALB/cJ urine, synthetic 6H6MH3O was shown to have no effect on uterine growth in BALB/cJ female mice, in contrast to its effect in C57BL/6J mice (Flanagan et al., 2011).

6H6MH3O is a highly unusual metabolite. Although sugars like D-glucose exist in aqueous solution in equilibrium among a variety of forms, they largely exist in equilibrium between the cyclic, chiral, alpha-D-, and beta-D-glucopyranose anomers. By contrast, 6H6MH3O exists in equilibrium between an acyclic, achiral tautomer and a cyclic, chiral tautomer (Antonov, 2014). To the best of our knowledge, the tautomerisation of 6H6MH3O is unique amongst known metabolites. Given the symmetry of the achiral ketone tautomer, it is probable that the chiral, hemi-ketal form in solution is a racemic mixture of the R and S enantiomers at C3.

The citric acid cycle metabolites succinate, 2-oxoglutarate and citrate all showed higher concentrations in the urine of FMO5 KO and WT mice as they aged from weeks 15 to 60. Increased concentrations of citric acid cycle intermediates have been reported previously in urine of 16-week-old NMRI mice compared with those aged 14 weeks (Li et al., 2013), as well as ERCC1d/- mice (Nevedomskaya et al., 2010). However, it should be noted that in the present study the urinary concentrations of citric acid cycle intermediates were independent of their plasma concentrations. This is because these metabolites can be reabsorbed into the tubular cells, based on the intracellular pH of the kidney, and hence alterations in their urinary concentrations could be in response to different age-related physiological factors, which may influence the intracellular pH of kidney tubular cells.

The concentrations of microbiota-related urinary metabolites, including hippurate, indoxylsulphate, phenylacetylglycine, trigonelline, and cinnamoylglycine, as well as aliphatic amines such as dimethylamine, trimethylamine, and trimethylamine *N*-oxide, were also altered with ageing. The aliphatic amines are produced from degradation of dietary precursors, such as choline or trimethylamine *N*-oxide, by the gut microbiota, (Fennema et al., 2016) and alteration of these metabolites suggests age-related changes in the activities or populations of the gut microbiome. Of particular interest is that at an earlier age (week 15–30), changes in urinary microbiome-related metabolites were different in WT, relative to FMO5 KO, mice. This was manifested by higher concentrations of cinnamoylglycine, 4-cresolglucuronide, 4-cresolsulphate, phenylacetylglycine and indoxylsulphate with age in WT mice, whereas no significant age-related changes in the concentrations of these metabolites were observed in FMO5 KO mice. This indicates that the composition and activity of the microbiome not only changes with ageing but it is also different in male FMO5 KO mice compared with male WT mice. These results are consistent with the recent findings of significant differences in gut microbiomes between FMO5 KO and WT mice by Scott et al (Scott et al., 2017) and their hypothesis that FMO5 has a role in sensing or responding to gut bacteria. Furthermore, we also observed decreased concentrations of short-chain fatty acids (SCFAs) and their glycine conjugates, including isovalerate, butyrylglycine and hexanoylglycine, in the urines of both FMO5 KO and WT mice over the course of ageing. Although we cannot directly infer causality, the reduction of SCFAs may also be associated with changes in the gut microbiome. These findings are in agreement with other studies demonstrating an age-related decrease in the abundance of SCFA producers in humans, and increases in the number of gut bacteria involved in aromatic amino-acid metabolism as well as facultative anaerobes and opportunistic pathogens (Rampelli et al., 2013).

Age-related changes were also observed in the urinary excretion of ascorbate. The level of ascorbate was increased from 15 to 30 weeks in both FMO5 KO and WT mice and then decreased in WT mice as they age from 30 to 45 weeks while it remained stable in KO mice. Ascorbate is known to be helpful in preventing or delaying the progression of ageing and age-related disease (Monacelli et al., 2017). Iwama et al. (2012) reported decreased ascorbate concentration in the urine of C57BL/6 mice as they aged from 6 to 30 months and suggested that ascorbate-synthesizing ability decreases over time are a key element in age-related diseases (Iwama et al., 2012).

Elevated urinary concentrations of taurine represent a distinctive ageing-related change observed only in WT mice. Urinary taurine was increased as WT mice aged from 15 to 30, 30 to 45 and overall from 15 to 60 weeks. Interestingly, taurine was also found to be a discriminator between KO and WT mice and was consistently at statistically significantly (FDR 0.1 adjusted) lower concentrations in FMO5 KO mice from 30 weeks of age onwards. Although taurine was detected by 2D ^1^H, ^13^C HSQC NMR experiments at low concentrations in both WT and FMO5 KO plasma samples, it was not possible to determine what changes, if any, occurred with ageing in plasma, as the low level signals in the ^1^H NMR spectra were obscured by much larger signals from glucose.

Increased excretion of taurine with ageing has been previously observed in dogs (Wang et al., 2007), male Sprague Dawley rats (Schnackenberg et al., 2007), and male Wistar-derived rats (Williams et al., 2005). Given that urinary taurine concentration is mainly regulated by renal reabsorption, the age-related increase of urinary taurine in WT mice may be caused by reduced renal reabsorption of taurine. In agreement with this hypothesis, preliminary desorption electrospray imaging mass spectrometry (DESI-MS) data comparing concentrations of taurine in the livers of male FMO5 KO and WT mice at week 30, showed significantly higher concentrations in the FMO5 KO mouse liver (Supplementary Figure 18).

Taurine is the most abundant, multifunctional amino acid, and plays an essential role in a large number of biological processes including bile acid conjugation, cellular osmoregulation, modulation of neurotransmitters, maintenance of calcium homeostasis, and antioxidation (Hayes and Sturman, 1981; Brosnan and Brosnan, 2006). Taurine is also well known for its protective effect against diabetes mellitus and the complications of diabetes, including retinopathy, nephropathy, neuropathy, atherosclerosis, and cardiomyopathy, as well as protective effects against other age-associated diseases (Ito et al., 2012). The increase in urinary excretion of taurine with ageing in WT mice relative to FMO5 KO mice indicates that taurine's protective function of anti-inflammation, immunomodulation and neuroprotection might be attenuated in WT mice and hence they are likely to be more susceptible to age-related diseases relative to FMO5 KO mice, a conclusion supported by our preliminary DESI-MS data (Supplementary Figure 18).

Another characteristic of the ageing WT mouse (week 60 vs. 45, Table 1) was increased urinary concentrations of 1-methylnicotinamide (1MNA). No such difference was observed in the FMO5 KO mice between these same two time points but 1MNA was increased in both WT and FMO5 KO mice with age overall between weeks 15 and 60 (Table 1). 1MNA is produced in the liver by nicotinamide *N*-methyltransferase (NNMT) by catalysis of the *N*-methylation of nicotinamide. Nicotinamide is a precursor of nicotinamide adenine dinucleotide (NAD) and nicotinamide adenine dinucleotide phosphate, which are known to be associated with longevity through the activity of NAD-consuming enzymes, such as sirtuins and poly (ADP-ribose) polymerases (Imai, 2011; Roth et al., 2013). 1MNA contributes to the regulation of intra- and extra-cellular concentrations of nicotinamide by mediating its excretion after *N*-methylation. The increased urinary excretion of 1MNA with age may therefore indicate a perturbation in the “NAD World” homeostasis (Robertson, 2005; Everard et al., 2011).

Age-related changes were also observed in the urinary excretion of creatinine. The urinary concentration of creatinine was increased from 15 to 30 weeks in both FMO5 KO and WT mice and then remained stable. In general, the excretion of creatinine can be influenced by factors such as the change of total muscle mass, dietary protein intake and glomerular filtration rate. In the present study, the higher concentration of urinary creatinine observed in week 30 relative to week 15 mice probably reflects the growth of the animals. Such age-related increases have been reported previously in male Wistar-derived rats (Williams et al., 2005), as well as male Sprague Dawley rats (Schnackenberg et al., 2007). Urinary excretion of creatinine was also increased in dogs between ages 5 and 9 years and decreased thereafter (Wang et al., 2007). Increases in creatinine excretion have been also noted in children as they progress to adulthood. In addition, a study on creatinine concentrations that surveyed a large US population, with ages ranging from 6 to 70 years, reported a gradual increase in urinary creatinine concentration up to an age of between 20 and 29 years, followed by a decline (Barr et al., 2005).

### Ageing-associated changes in plasma metabolite profiles

Unlike urine, metabolic profiles of plasma for both FMO5 KO and WT mice were more constant up to week 45 and the largest number of statistically significant metabolite changes was observed as the mice aged from 45 to 60 weeks in both cases (Table 2).

In plasma, the main metabolic signature of ageing includes alterations in the concentrations of metabolites associated with amino-acid and fatty-acid metabolism and the citric acid cycle (Table 2).

Higher concentration of amino acids including glutamine and the BCAA leucine, isoleucine and valine were observed in the plasma of male FMO5 KO and WT mice with ageing. It is likely that the elevation of amino acids in the plasma with increased age is caused by decreased rates of transamination and then subsequent oxidation of their carbon skeletons in the citric acid cycle.

## Conclusion

Significant changes in metabolite profiles with ageing were identified in both the urine and plasma of male WT and FMO5 KO mice through the use of an NMR-based metabonomic approach. Some metabolites showed similar patterns of changes with age, regardless of genetic background. However, we also observed different age-related metabolic changes between WT and FMO5 KO mice, indicating the impact of the genetic modification on ageing. The metabolite changes observed and the differences in ageing profiles between the WT and FMO5 KO genotypes reflect both general ageing process in both genotypes and specific changes that are characteristic of the slow metabolic-ageing phenotype of the FMO5 KO mouse (Gonzalez Malagon et al., 2015; Scott et al., 2017). The identification of these metabolites that change with ageing will help understand the processes of ageing in these two mouse genotypes and the differences between them, including the important impact of the gut microbiome and its interactions with the host genome, and, as such, we hope this work will in future generate new ideas and understanding to extend healthy human lifespan.

## Author contributions

JE, ES, and IP: Conceived the experiments; DorsaV, JE, ES, and IP: Designed the experiments; DorsaV, JE, IP, FS, ES, NS, ZT, and SV: Conducted the experiments; KV and colleagues: Wrote the statistical analysis software; DorsaV, DornaV, and JE: Analyzed the data; DorsaV and JE: Wrote the paper. All authors reviewed the paper.

### Conflict of interest statement

The authors declare that the research was conducted in the absence of any commercial or financial relationships that could be construed as a potential conflict of interest.

## Supplementary material

The original NMR spectral data files for the C57BL/6J mice will be deposited in MetaboLights at https://www.ebi.ac.uk/metabolights.
