# Supplementary material for: Metabolic Biomarkers of Ageing in C57BL/6J Wild-Type and Flavin-Containing Monooxygenase 5 (FMO5)-Knockout Mice
Source: Front Mol Biosci. 2018 Apr 9;5:28. doi: 10.3389/fmolb.2018.00028 (PMC5900034; doi:10.3389/fmolb.2018.00028)
Supplement: Supplementary file 1 [file DataSheet1.docx]

**Supplementary Information: Metabolic Biomarkers of Ageing in C57BL/6J Wild-Type and Flavin-containing Monooxygenase 5 (FMO5)-Knockout Mice**

Authors: Dorsa Varshavi^1a^, Flora. H Scott^2b^, Dorna Varshavi^1^, Sunil Veeravalli^2^, Ian R. Phillips^2, 3^, Kirill Veselkov^4^, Nicole Strittmatter^4^, Zoltan Takats^4^, Elizabeth A. Shephard^2^, and Jeremy R. Everett^1*^

^1^ Medway Metabonomics Research Group,

University of Greenwich,

Chatham Maritime,

Kent, ME4 4TB

United Kingdom

^2^ Institute of Structural and Molecular Biology,

University College London,

London, WC1E 6BT

United Kingdom

^3^ School of Biological and Chemical Sciences,

Queen Mary University of London,

London, E1 4NS

United Kingdom

^4^ Department of Surgery and Cancer,

Faculty of Medicine,

Imperial College,

London, SW7 2AZ

United Kingdom

^a^ Current Address:

School of Biosciences,
University of Birmingham,
Edgbaston,
Birmingham, B15 2TT
United Kingdom

^b^ Current Address

Imperial Centre for Translational and Experimental Medicine,

Imperial College,

Hammersmith Campus,

72 Du Cane Road,

Shepherds Bush

London W12 0UQ

United Kingdom

* Author for Correspondence

Telephone: +44 (0)208 331 8323

Email: [j.r.everett@greenwich.ac.uk](mailto:j.r.everett@greenwich.ac.uk)

**The Identification of the hydroxy-ketone and hemi-ketal tautomers of 6-hydroxy-6-methyl-heptan-3-one**

The identification of the hydroxyl-ketone tautomer of 6-hydroxy-6-methyl-heptan-3-one was made as follows. In a 600 MHz ^1^H NMR spectrum from a male, week 30, FMO5 KO mouse a sharp, methyl (Me-7 and C6-Me) singlet (s) signal at 1.209 with an HSQC to 30.4 (C7 and C6-Me) showed HMBC signals to 30.3 (cross-methyl C6-Me to C7 and back), 39.2 (C5) and 73.6 (C6), establishing the left-hand side of the molecule (see Supplementary Figure 5). The C5 methylene protons at 1.739 (2^nd^ order multiplet, shift from JRES) had an HSQC to 39.3 (C5) with a strong COSY to 2.620, 40.0 (C4) and HMBC connectivities to 30.4 (C6-Me and C7), 39.9 (C4), 73.6 (C6) and 222.3 (C3). The C4 methylene protons at 2.620 had an HSQC to 40.0 and HMBC connectivities to 39.2 (C5) and 222.4 (C3), thus connecting the middle portion of the metabolite to the left-hand side. The methyl group at 1.015 (triplet, t, 7.3 Hz), 10.1 (C1) had a strong COSY to 2.598 (quartet, q, ca 7.4 Hz), 38.6 (C2) and HMBC connectivities to 38.7 (C2) and 222.2 (C3), thus completing the identification of the hydroxy-ketone tautomer of this metabolite (see also Supplementary Table 3).

Since the linear, achiral, hydroxy-ketone tautomer should exist in equilibrium with the cyclic, chiral, hemi-ketal tautomer (see structure panel above), a search was made in a high-sensitivity HMBC spectrum of the same male, week 30, FMO5 KO urine for the C6 hemi-ketal signals that were predicted to be at ca. 86.5, by reference to the corresponding data for the authentic hemi-ketal of the homologous metabolite, 5-hydroxy-5-methyl-hexan-2-one (synthesized by Enamine, Ukraine). Reassuringly, HMBC signals were observed from 1.367 (s, C6-Me) to 86.2 (C6), 39.0 (C5) and 30.3 (weak, cross-methyl) and from 1.197 (s, C7) to 31.9 (cross-methyl) and 86.2 (C6), exactly as would be predicted from the data for the hemi-ketal of authentic 5-hydroxy-5-methyl-hexan-2-one (1.36, 29.7 (C5-Me); 1.21, 30.3 (C6); 39.4 (C4) and 86.5 (C5), all data for buffer/TSP; note the numbering differences for the hexanone with respect to the heptanone). The two C6 methyl groups are non-equivalent in the hemiketal tautomers and even show a characteristic but weak ^4^J_H,H_ cross peak in the FMO5 KO urine COSY between the resonances at 1.370 and 1.205 (see also Supplementary Table 3).

UPLC-MS data on a sample of authentic Enamine 6-hydroxy-6-methyl-heptan-3-one showed one major component eluting at 4.6 min. The high-resolution, positive-ion electrospray mass spectrum of this component showed ions at *m/z* 127.1119 (C_8_H_15_O, 0.8 ppm error), corresponding to [M+H-H_2_O]^+^, together with ions at *m/z* 109.1013, 67.0547 and 57.034. No protonated molecular-ion signal was observed. The corresponding UPLC-MS data on a week 15, WT mouse urine sample showed a component eluting at 4.7 min with significant peaks at *m/z* 127.1116 (C_8_H_15_O, 1.6 ppm error), 109.1014, 67.0540 and 57.0339. Note that the mass error figures quoted are corrected for the mass of the electron. The commercial spectrometer software fails to do this, which is critical for low-mass metabolites such as this, and gives erroneous mass error figures.

**Supplementary Figure 1.**  At bottom a ‘heat map’ display of the 600 MHz urine ^1^H NMR spectra of FMO5 KO mice aged 30 weeks from batch 2 (top) and batch 1 (bottom). Red and blue elements in the spectra indicate NMR signals that are more intense, or less intense, respectively, than the median signal intensity for all the samples. At top, the corresponding ANOVA plot for these samples, showing positive peaks for those metabolite signals that are more intense in batch 2, and negative peaks for those metabolite signals that are less intense. The signals are colour coded by the *p* value from the ANOVA analysis. In this case a false discovery rate cut-off of 10% was used and the threshold *p*-value for significant difference was calculated as 0.00079, corresponding to those signals with colouring to the ‘red side’ of light blue. The lack of any significantly discriminating metabolites highlights the similarity between these two different groups of FMO5 KO mice.


**Supplementary Figure 2.** At bottom a ‘heat map’ display of the 600 MHz urine ^1^H NMR spectra of WT mice aged 30 weeks from batch 2 (top) and batch 1 (bottom)..Red and blue elements in the spectra indicate NMR signals that are more intense, or less intense, respectively, than the median signal intensity for all the samples. At top, the corresponding ANOVA plot for these samples, showing positive peaks for those metabolite signals that are more intense in batch 2, and negative peaks for those metabolite signals that are less intense. The signals are colour coded by the *p* value from the ANOVA analysis. In this case a false discovery rate cut-off of 10% was used and the threshold *p*-value for significant difference was calculated as 6 x 10^-7^,corresponding to signals with colouring to the ‘red side’ of light blue. The lack of any significantly discriminating metabolites highlights the similarity between these two different groups of FMO5 KO mice.

**Supplementary Figure 3.**  At bottom a ‘heat map’ display of the 600 MHz plasma ^1^H NMR spectra of FMO5 KO mice aged 30 weeks from batch 1 (top) and batch 2 (bottom), Red and blue elements in the spectra indicate NMR signals that are more intense, or less intense, respectively, than the median signal intensity for all the samples. At top, the corresponding ANOVA plot for these samples, showing positive peaks for those metabolite signals that are more intense in batch 1, and negative peaks for those metabolite signals that are less intense. The signals are colour coded by the *p* value from the ANOVA analysis. In this case a false discovery rate cut-off of 10% was used and the threshold *p*-value for significant difference was calculated as 0.00007, corresponding to those signals with colouring to the ‘red side’ of light blue.

**Supplementary Figure 4.**  At bottom a ‘heat map’ display of the 600 MHz plasma ^1^H NMR spectra of WT mice aged 30 weeks from batch 1 (top) and batch 2 (bottom). Red and blue elements in the spectra indicate NMR signals that are more intense, or less intense, respectively, than the median signal intensity for the all samples. At top, the corresponding ANOVA plot for these samples, showing positive peaks for those metabolite signals that are more intense in batch 1, and negative peaks for those metabolite signals that are less intense. The signals are colour coded by the *p* value from the ANOVA analysis. In this case a false discovery rate cut-off of 10% was used and the threshold *p*-value for significant difference was calculated as 0.00008, corresponding to those signals with colouring to the ‘red side’ of light blue.

**
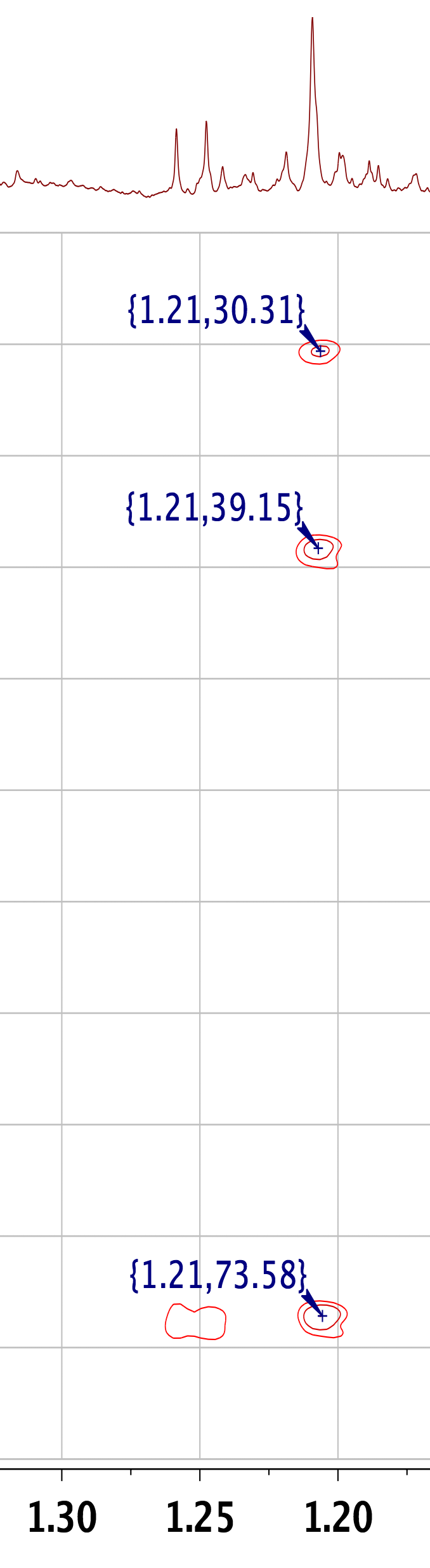
**

**Supplementary Figure 5.** A strip of the contour plot of the 600 MHz ^1^H, ^13^C 2D HMBC NMR spectrum of the urine of a week 30 FMO5 KO mouse, underneath the corresponding portion of the 1D ^1^H NMR spectrum. The sharp, methyl singlet signal at 1.209 (Me-7 and C6-Me) shows strong HMBC cross-peaks to 30.3 (cross-methyl C6-Me to C7 and back), 39.2 (C5) and 73.6 (C6),

**Supplementary Figure 6.** At bottom a ‘heat map’ display of the 600 MHz urine ^1^H NMR spectra of WT mice at week 30 age (top four strips) vs the corresponding spectra of WT mice at week 15 age (bottom 4 strips). Red and blue elements in the spectra indicate NMR signals that are more intense, or less intense, respectively, than the median signal intensity for the whole cohort. At top, the corresponding ANOVA plot for this cohort, showing positive peaks for those metabolite signals that are more intense at week 30 than at week 15, and negative peaks for those metabolite signals that are less intense. The signals are colour coded by the *p* value from the ANOVA analysis. In this case a false discovery rate cut-off of 10% was used and the threshold *p*-value for significant difference was calculated as 0.01569, corresponding to those signals with colouring to the ‘red side’ of light blue.


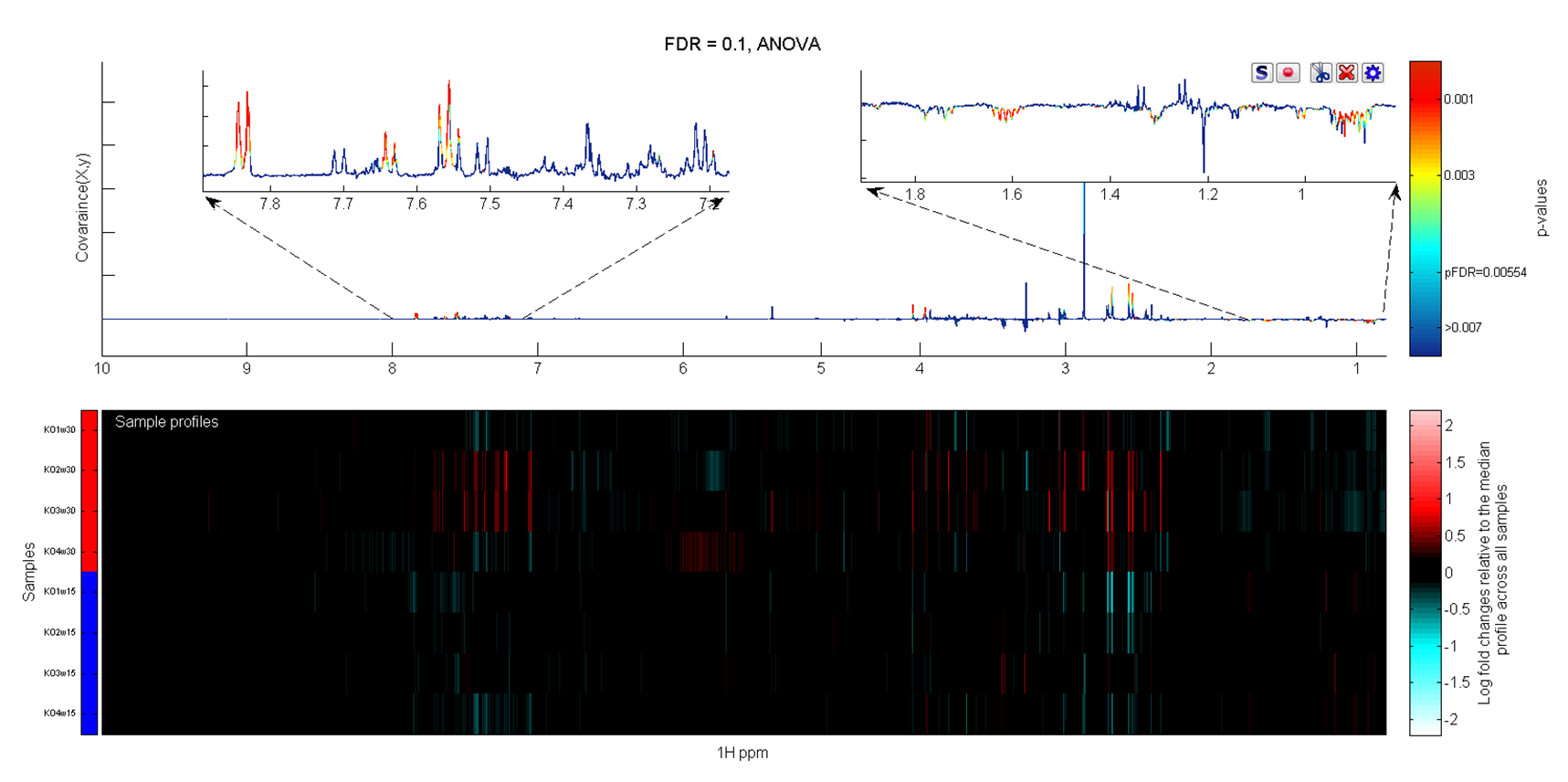


**Supplementary Figure 7.** At bottom a ‘heat map’ display of the 600 MHz urine ^1^H NMR spectra of FMO5 KO mice at week 30 age (top four strips) vs the corresponding spectra of KO mice at week 15 age (bottom 4 strips). Red and blue elements in the spectra indicate NMR signals that are more intense, or less intense, respectively, than the median signal intensity for the whole cohort. At top, the corresponding ANOVA plot for this cohort, showing positive peaks for those metabolite signals that are more intense at week 30 than at week 15, and negative peaks for those metabolite signals that are less intense. The signals are colour coded by the *p* value from the ANOVA analysis. In this case a false discovery rate cut-off of 10% was used and the threshold *p*-value for significant difference was calculated as 0.00554, corresponding to those signals with colouring to the ‘red side’ of light blue.

**Supplementary Figure 8.** At bottom a ‘heat map’ display of the 600 MHz ^1^H NMR spectra of the urine of WT mice at week 45 age (top four strips) vs the corresponding spectra of WT mice at week 30 age (bottom 4 strips). Red and blue elements in the spectra indicate NMR signals that are more intense, or less intense, respectively, than the median signal intensity for the whole cohort. At top, the corresponding ANOVA plot for this cohort, showing positive peaks for those metabolite signals that are more intense at week 45 than at week 30, and negative peaks for those metabolite signals that are less intense. The signals are colour coded by the *p* value from the ANOVA analysis. In this case a false discovery rate cut-off of 10% was used and the threshold *p*-value for significant difference was calculated as 0.01191, corresponding to those signals with colouring to the ‘red side’ of light blue.

**Supplementary Figure 9.** At bottom a ‘heat map’ display of the 600 MHz urine ^1^H NMR spectra of FMO5 KO mice at week 45 age (top five strips) vs the corresponding spectra of KO mice at week 30 age (bottom 4 strips). Red and blue elements in the spectra indicate NMR signals that are more intense, or less intense, respectively, than the median signal intensity for the whole cohort. At top, the corresponding ANOVA plot for this cohort, showing positive peaks for those metabolite signals that are more intense at week 45 than at week 30, and negative peaks for those metabolite signals that are less intense. The signals are colour coded by the *p* value from the ANOVA analysis. In this case a false discovery rate cut-off of 10% was used and the threshold *p*-value for significant difference was calculated as 0.00334, corresponding to those signals with colouring to the ‘red side’ of light blue.


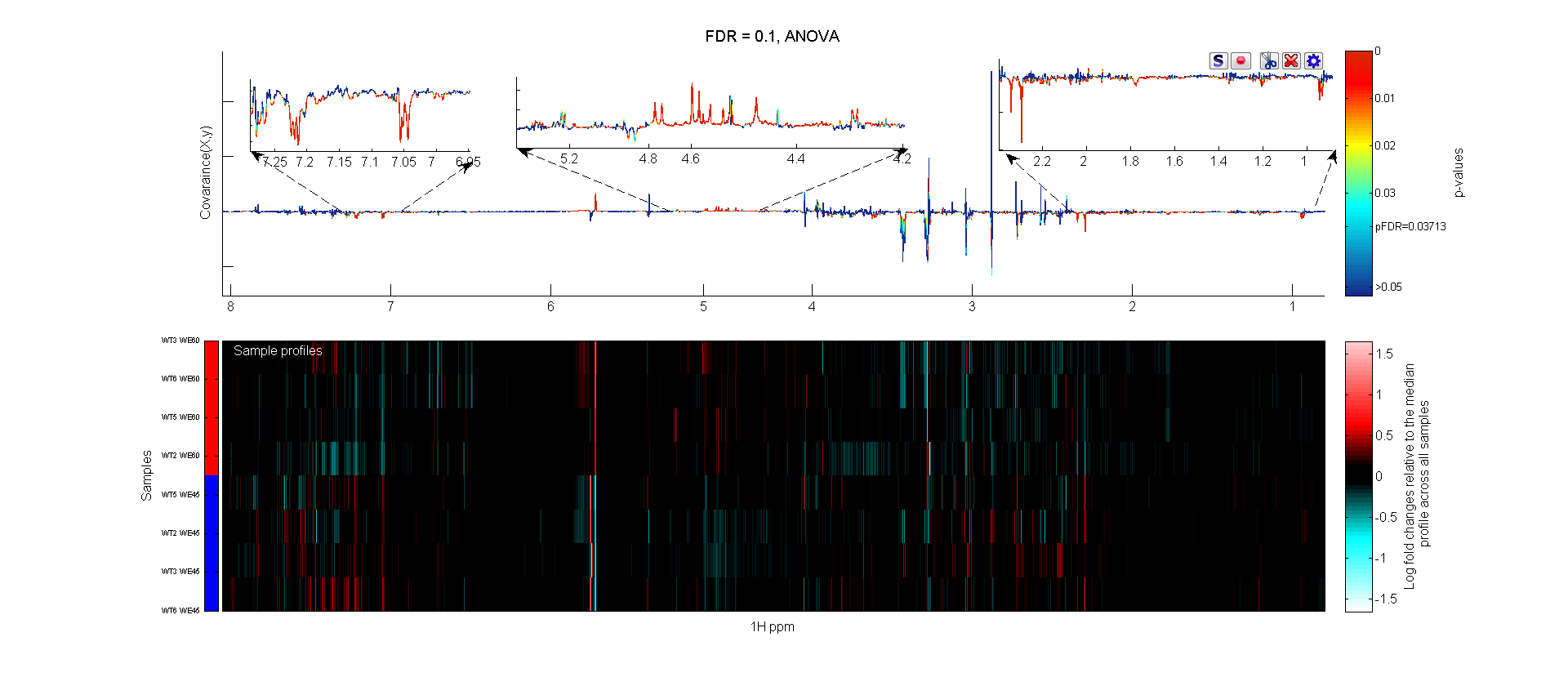


**Supplementary Figure 10.** At bottom a ‘heat map’ display of the 600 MHz urine ^1^H NMR spectra of WT mice at week 60 age (top four strips) vs the corresponding spectra of WT mice at week 45 age (bottom 4 strips). Red and blue elements in the spectra indicate NMR signals that are more intense, or less intense, respectively, than the median signal intensity for the whole cohort. At top, the corresponding ANOVA plot for this cohort, showing positive peaks for those metabolite signals that are more intense at week 60 than at week 45, and negative peaks for those metabolite signals that are less intense. The signals are colour coded by the *p* value from the ANOVA analysis. In this case a false discovery rate cut-off of 10% was used and the threshold *p*-value for significant difference was calculated as 0.03713, corresponding to those signals with colouring to the ‘red side’ of light blue.


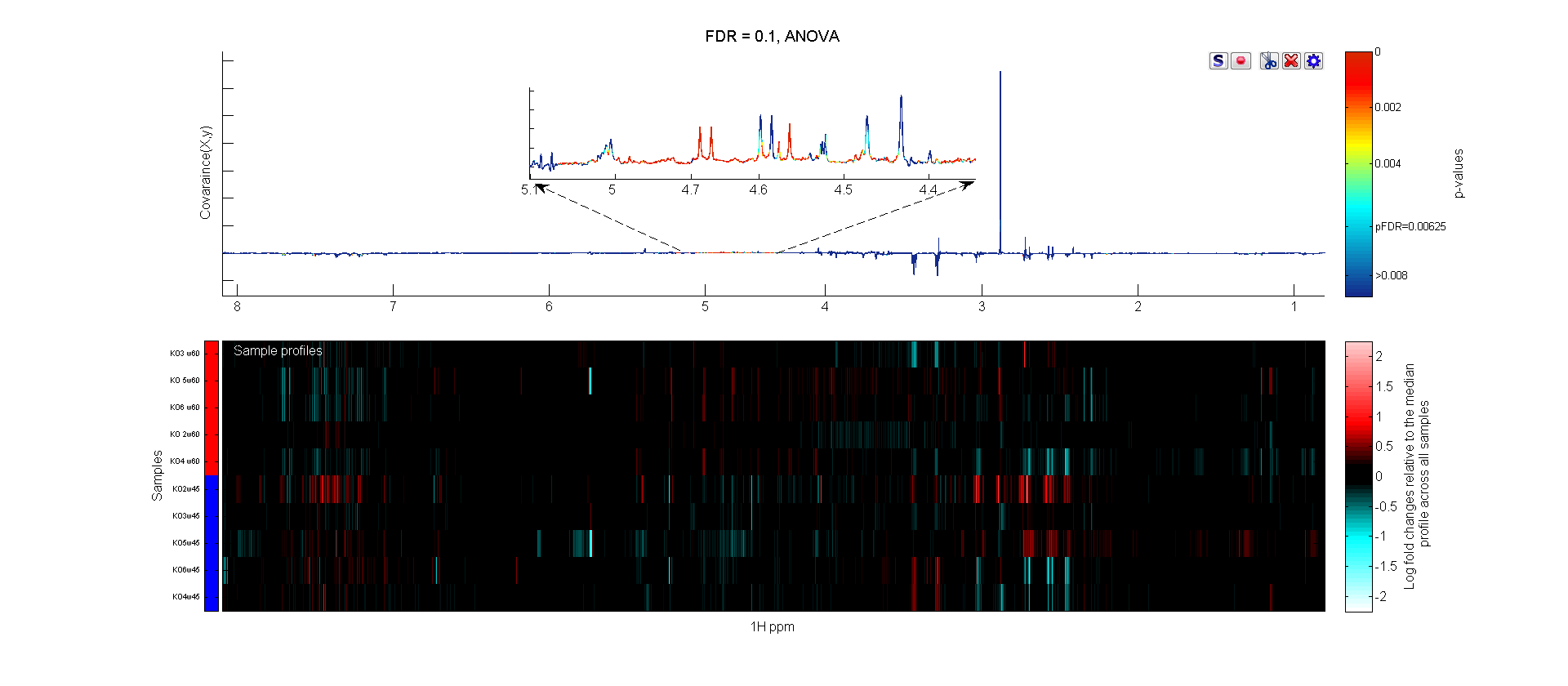


**Supplementary Figure 11.** At bottom a ‘heat map’ display of the 600 MHz urine ^1^H NMR spectra of FMO5 KO mice at week 60 age (top five strips) vs the corresponding spectra of KO mice at week 45 age (bottom 4 strips). Red and blue elements in the spectra indicate NMR signals that are more intense, or less intense, respectively, than the median signal intensity for the whole cohort. At top, the corresponding ANOVA plot for this cohort, showing positive peaks for those metabolite signals that are more intense at week 60 than at week 45, and negative peaks for those metabolite signals that are less intense. The signals are colour coded by the *p* value from the ANOVA analysis. In this case a false discovery rate cut-off of 10% was used and the threshold *p*-value for significant difference was calculated as 0.00626, corresponding to those signals with colouring to the ‘red side’ of light blue.


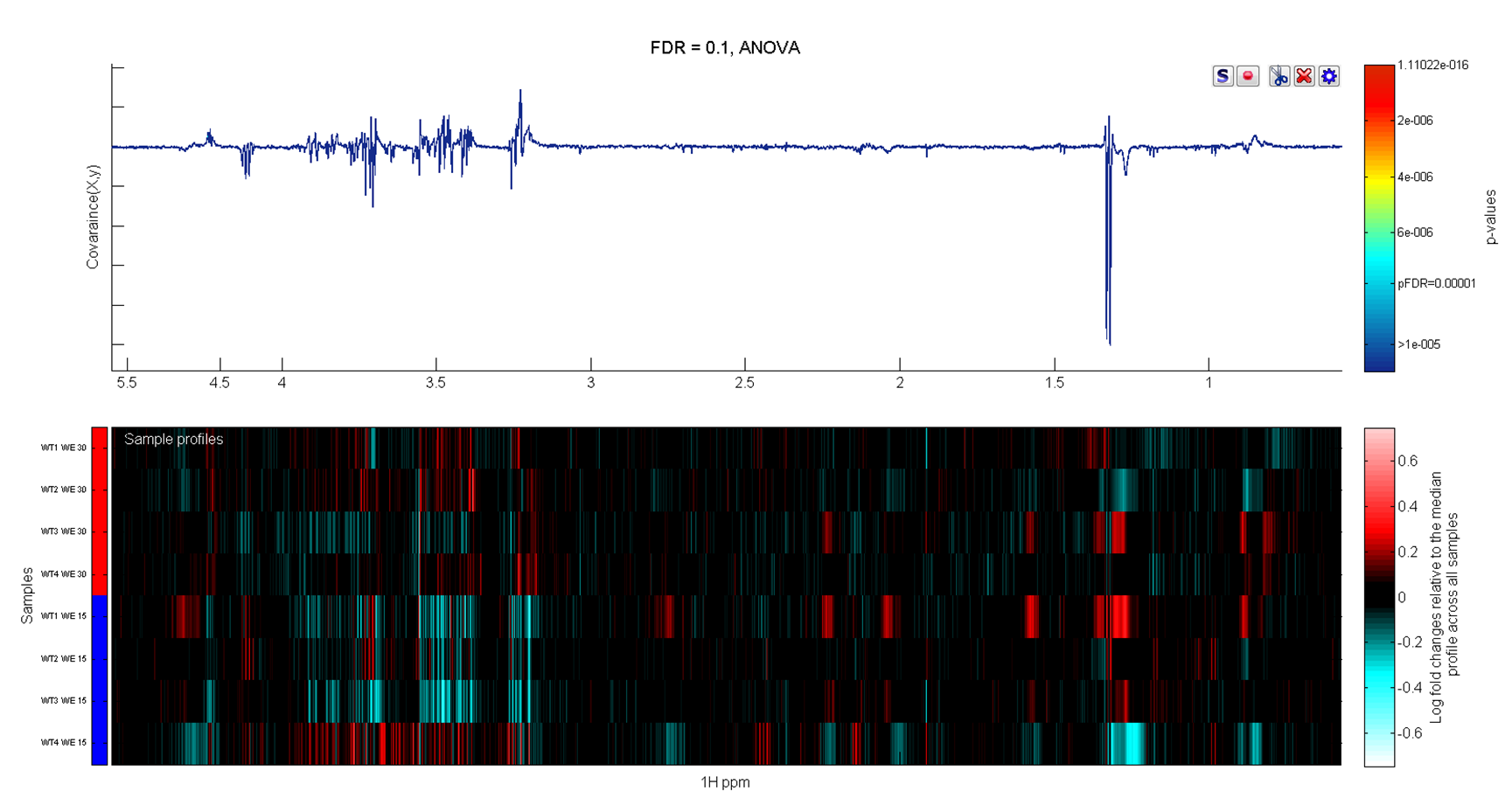


**Supplementary Figure 12.** At bottom a ‘heat map’ display of the 600 MHz plasma ^1^H NMR spectra of WT mice at week 30 age (top four strips) vs the corresponding spectra of WT mice at week 15 age (bottom 4 strips). Red and blue elements in the spectra indicate NMR signals that are more intense, or less intense, respectively, than the median signal intensity for the whole cohort. At top, the corresponding ANOVA plot for this cohort, showing positive peaks for those metabolite signals that are more intense at week 30 than at week 15, and negative peaks for those metabolite signals that are less intense. The signals are colour coded by the *p* value from the ANOVA analysis. In this case a false discovery rate cut-off of 10% was used and the threshold *p*-value for significant difference was calculated as 0.00001, corresponding to those signals with colouring to the ‘red side’ of light blue.


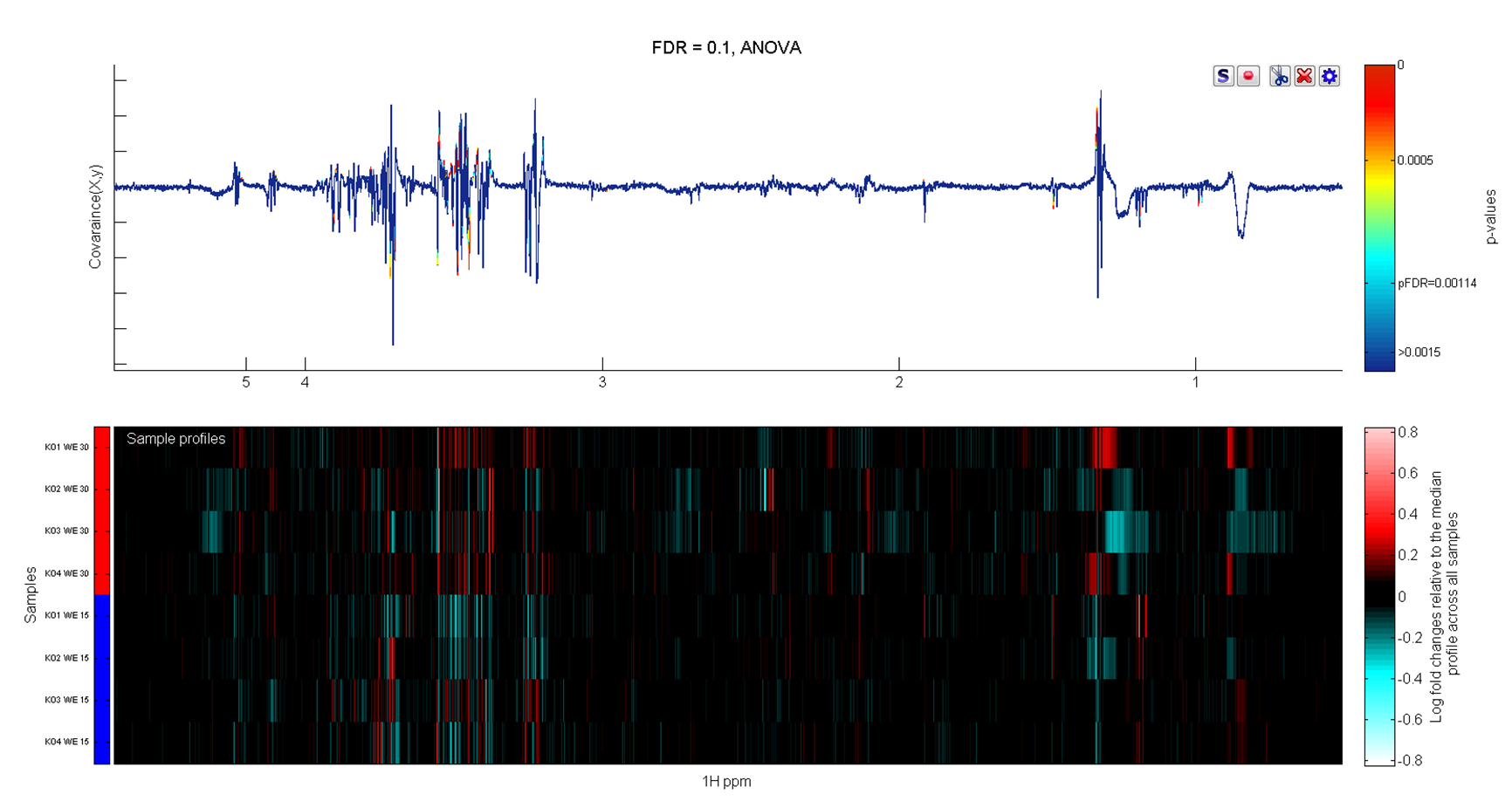


**Supplementary Figure 13**. At bottom a ‘heat map’ display of the 600 MHz plasma ^1^H NMR spectra of FMO5 KO mice at week 30 age (top four strips) vs the corresponding spectra of KO mice at week 15 age (bottom 4 strips). Red and blue elements in the spectra indicate NMR signals that are more intense, or less intense, respectively, than the median signal intensity for the whole cohort. At top, the corresponding ANOVA plot for this cohort, showing positive peaks for those metabolite signals that are more intense at week 30 than at week 15, and negative peaks for those metabolite signals that are less intense. The signals are colour coded by the *p* value from the ANOVA analysis. In this case a false discovery rate cut-off of 10% was used and the threshold *p*-value for significant difference was calculated as 0.00114, corresponding to those signals with colouring to the ‘red side’ of light blue.

**Supplementary Figure 14.** At bottom a ‘heat map’ display of the 600 MHz plasma^1^H NMR spectra of WT mice at week 45 age (top four strips) vs the corresponding spectra of WT mice at week 30 age (bottom 4 strips). Red and blue elements in the spectra indicate NMR signals that are more intense, or less intense, respectively, than the median signal intensity for the whole cohort. At top, the corresponding ANOVA plot for this cohort, showing positive peaks for those metabolite signals that are more intense at week 45 than at week 30, and negative peaks for those metabolite signals that are less intense. The signals are colour coded by the *p* value from the ANOVA analysis. In this case a false discovery rate cut-off of 10% was used and the threshold *p*-value for significant difference was calculated as 0.00154, corresponding to those signals with colouring to the ‘red side’ of light blue.

**Supplementary Figure 15.** At bottom a ‘heat map’ display of the 600 MHz plasma ^1^H NMR spectra of FMO5 KO mice at week 45 age (top four strips) vs the corresponding spectra of KO mice at week 30 age (bottom 4 strips). Red and blue elements in the spectra indicate NMR signals that are more intense, or less intense, respectively, than the median signal intensity for the whole cohort. At top, the corresponding ANOVA plot for this cohort, showing positive peaks for those metabolite signals that are more intense at week 45 than at week 30, and negative peaks for those metabolite signals that are less intense. The signals are colour coded by the *p* value from the ANOVA analysis. In this case a false discovery rate cut-off of 10% was used and the threshold *p*-value for significant difference was calculated as 0.00170, corresponding to those signals with colouring to the ‘red side’ of light blue.


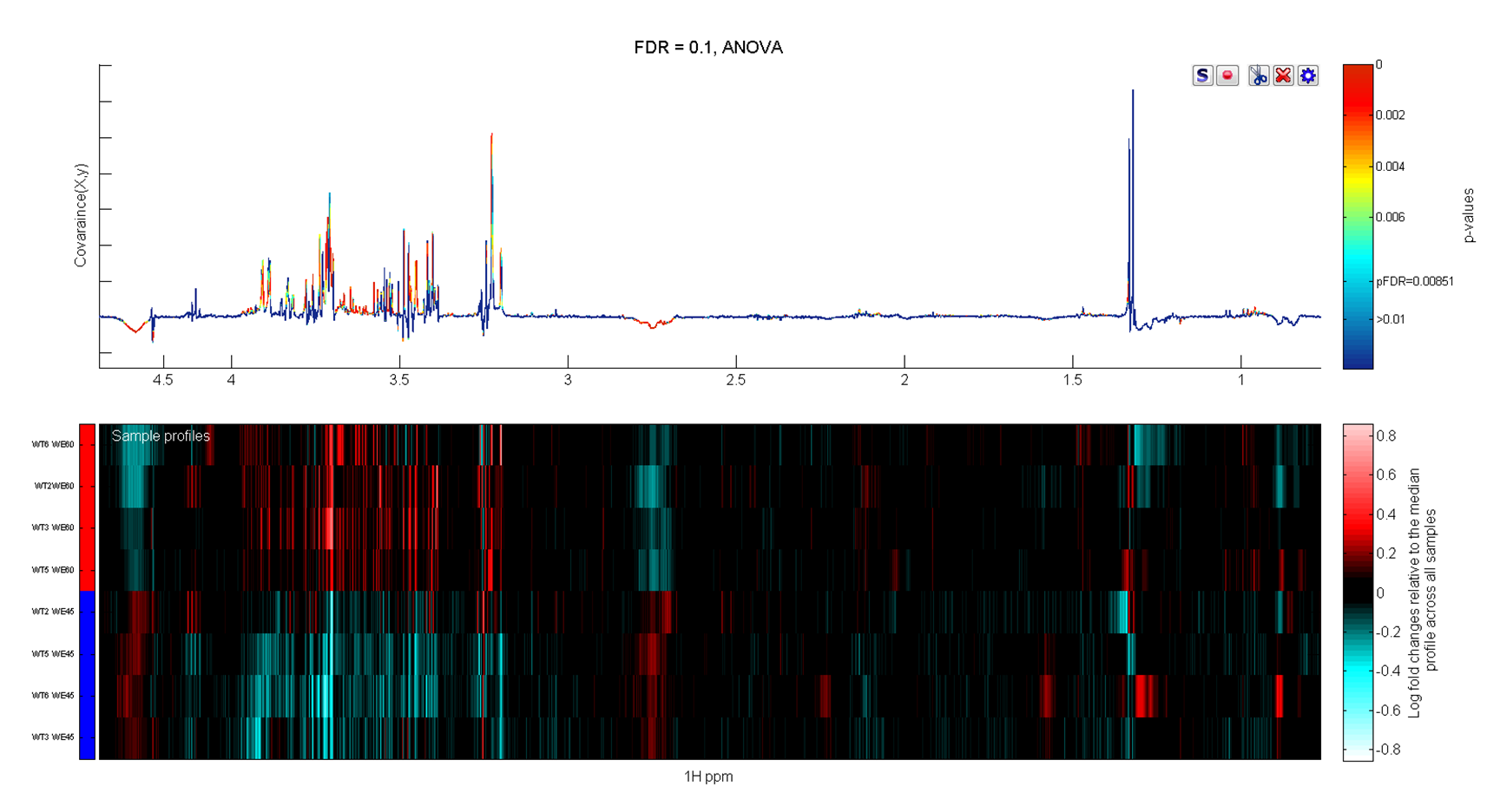


**Supplementary Figure 16.** At bottom a ‘heat map’ display of the 600 MHz plasma ^1^H NMR spectra of WT mice at week 60 age (top four strips) vs the corresponding spectra of WT mice at week 45 age (bottom 4 strips). Red and blue elements in the spectra indicate NMR signals that are more intense, or less intense, respectively, than the median signal intensity for the whole cohort. At top, the corresponding ANOVA plot for this cohort, showing positive peaks for those metabolite signals that are more intense at week 60 than at week 45, and negative peaks for those metabolite signals that are less intense. The signals are colour coded by the *p* value from the ANOVA analysis. In this case a false discovery rate cut-off of 10% was used and the threshold *p*-value for significant difference was calculated as 0.00051, corresponding to those signals with colouring to the ‘red side’ of light blue.

**
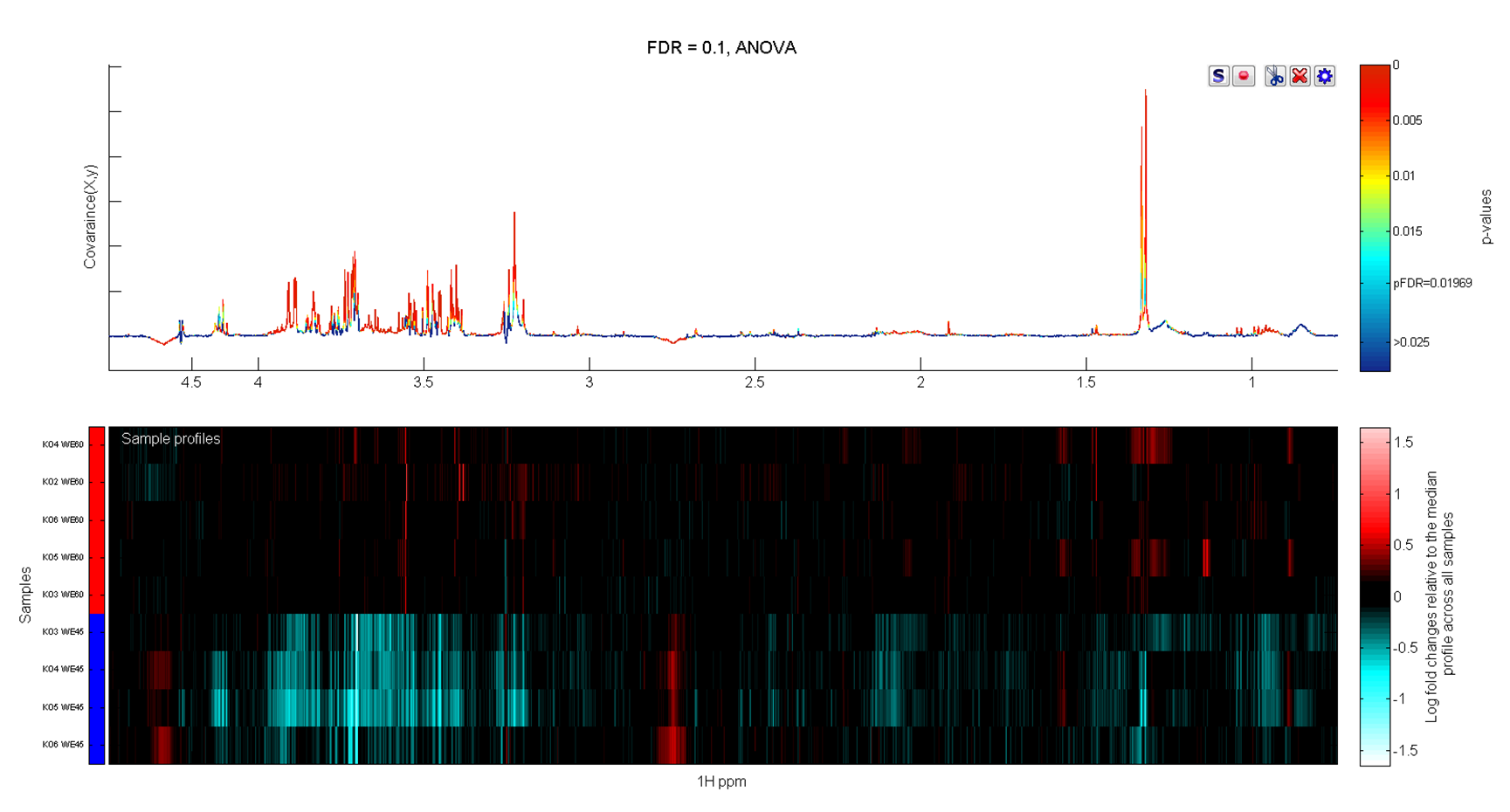
**

**Supplementary Figure 17.** At bottom a ‘heat map’ display of the 600 MHz plasma ^1^H NMR spectra of FMO5 KO mice at week 60 age (top five strips) vs the corresponding spectra of KO mice at week 45 age (bottom 4 strips). Red and blue elements in the spectra indicate NMR signals that are more intense, or less intense, respectively, than the median signal intensity for the whole cohort. At top, the corresponding ANOVA plot for this cohort, showing positive peaks for those metabolite signals that are more intense at week 60 than at week 45, and negative peaks for those metabolite signals that are less intense. The signals are colour coded by the *p* value from the ANOVA analysis. In this case a false discovery rate cut-off of 10% was used and the threshold *p*-value for significant difference was calculated as 0.01969, corresponding to those signals with colouring to the ‘red side’ of light blue.

**Supplementary Figure 18.** Negative ion DESI MS imaging of mouse liver: a) integrated ion image of liver tissues of KO and WT mice, the two left-hand-side tissue sections are from week 30 FMO5 KO mice, the centre and two right-hand-side tissue sections are from week 30 WT mice; b) DESI-MS ion image of taurine M-H^-^ ion at *m/z* 124.007; c) univariate analysis demonstrating higher concentrations of taurine in the liver of KO mice (red points) relative to WT mice (blue points).

**Supplementary Figure 19:** A superimposition of the low frequency region of the 600 MHz urine ^1^H NMR spectra of all the wildtype mice at weeks 15 and 60.

**Supplementary Figure 20:** A superimposition of the high frequency region of the 600 MHz urine ^1^H NMR spectra of all the wildtype mice at weeks 15 and 60.


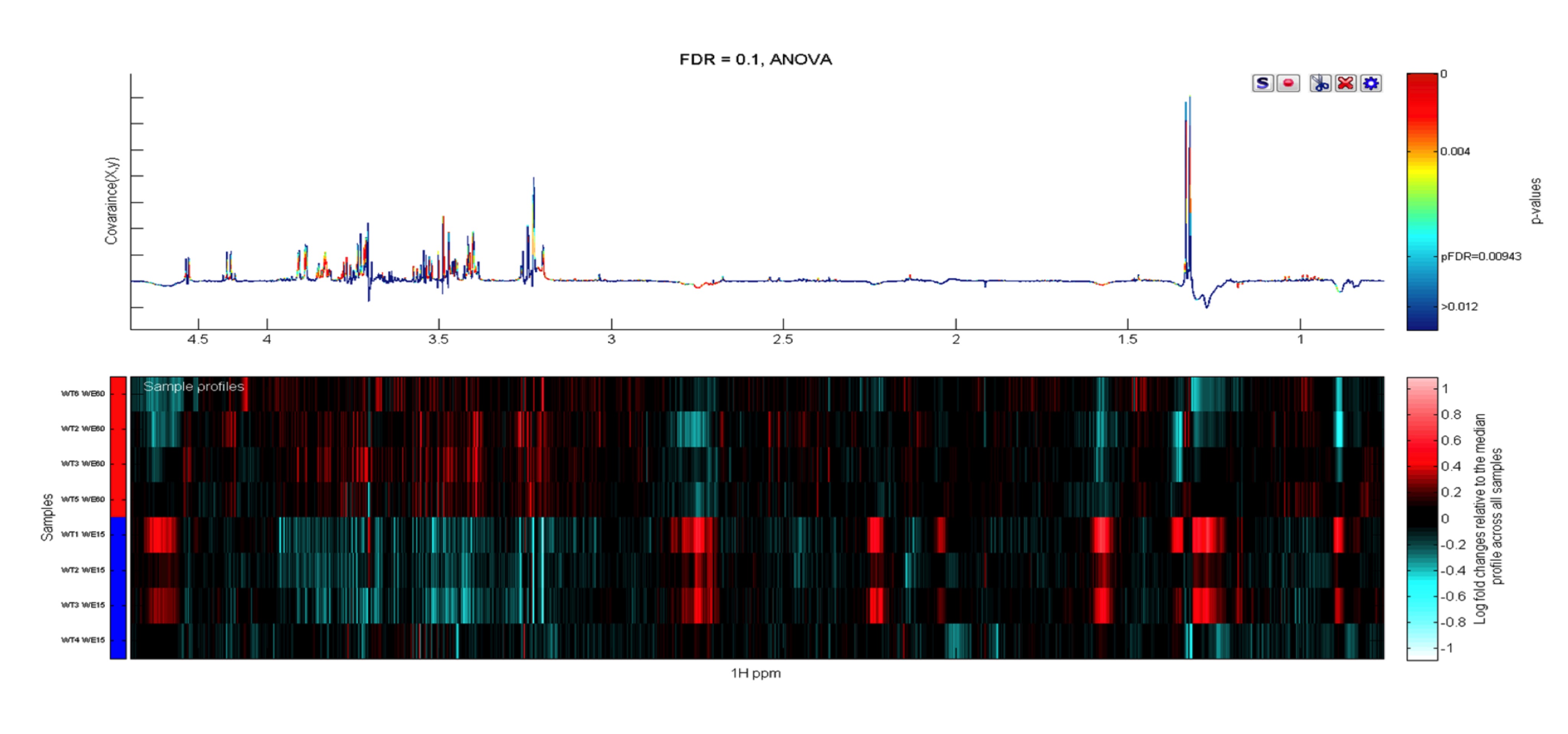


**Supplementary Figure 21.** At bottom a ‘heat map’ display of the 600 MHz plasma ^1^H NMR spectra of male WT mice at week 60 age (top four strips) vs the corresponding spectra of WT mice at week 15 age (bottom 4 strips). Red and blue elements in the spectra indicate NMR signals that are more intense, or less intense, respectively, than the median signal intensity for the whole cohort. At top, the corresponding ANOVA plot for this cohort, showing positive peaks for those metabolite signals that are more intense at week 60 than at week 15, and negative peaks for those metabolite signals that are less intense. The signals are colour coded by the *p*-value from the ANOVA analysis. In this case a false discovery rate cut-off of 10% was used and the threshold *p*-value for significant difference was calculated as 0.0094, corresponding to those signals with colouring to the ‘red side’ of light blue.


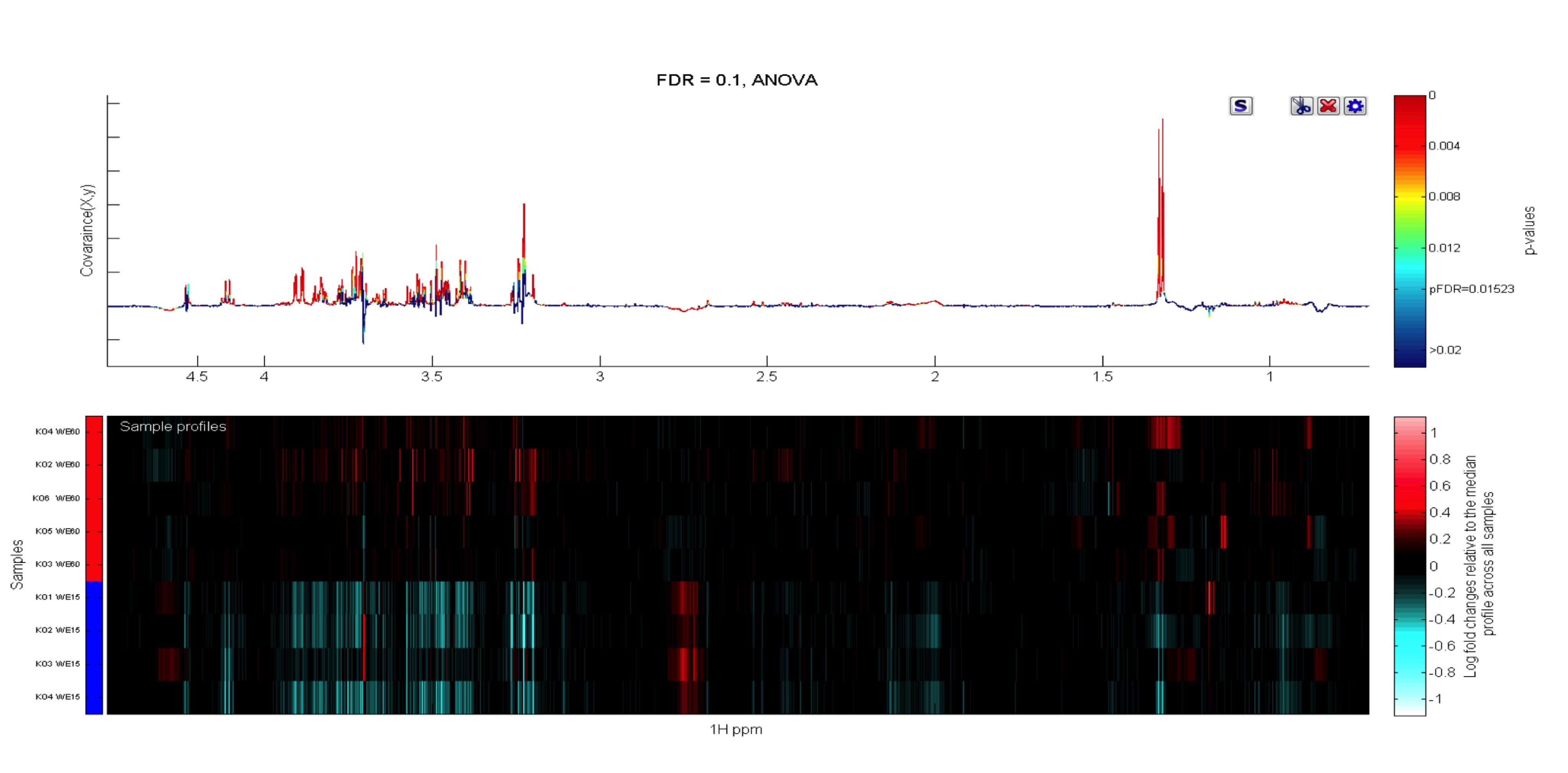
**Supplementary Figure 22.** At bottom a ‘heat map’ display of the 600 MHz plasma ^1^H NMR spectra of male FMO5 KO mice at week 60 age (top four strips) vs the corresponding spectra of KO mice at week 15 age (bottom 4 strips). Red and blue elements in the spectra indicate NMR signals that are more intense, or less intense, respectively, than the median signal intensity for the whole cohort. At top, the corresponding ANOVA plot for this cohort, showing positive peaks for those metabolite signals that are more intense at week 60 than at week 15, and negative peaks for those metabolite signals that are less intense. The signals are colour coded by the *p*-value from the ANOVA analysis. In this case a false discovery rate cut-off of 10% was used and the threshold *p*-value for significant difference was calculated as 0.015, corresponding to those signals with colouring to the ‘red side’ of light blue.

**Supplementary Table 1: data acquisition and processing parameters for 2D NMR spectra of urine samples from an FMO5 knockout mouse at week 30**

| **Parameter** | **JRES** | **COSY** | **TOCSY** | **HSQC** | **HMBC** |
| --- | --- | --- | --- | --- | --- |
| F2 spectral width in Hz | 12,019 | 6,009 | 6,128 | 9,615 | 6,203 |
| F1 spectral width in Hz | 50 | 6,004 | 6,126 | 30,187 | 33,523 |
| data points in t2 | 16,384 | 4,096 | 2,048 | 1,024 | 2,048 |
| spectral size in F2 | 32,768 | 8,192 | 4,096 | 4,096 | 4,096 |
| increments in t1 | 120 | 256 | 512 | 400 | 400 |
| spectral size in F1 | 1,024 | 4,096 | 4,096 | 2,048 | 2,048 |
| number of scans | 32 | 64 | 32 | 64 | 64 |
| relaxation delay (s) | 2.00 | 2.00 | 1.50 | 2.00 | 2.00 |
| apodisation | sine bell in t1 and t2 with first point correction in t1 | sine bell squared in t2, sine square and first point correction in t1 | sine bell squared in t2, sine square and first point correction in t1 | sine bell squared in t2, sine square with first point correction in t1 | sine bell in t1 and t2 with first point correction in t1 |
| Bruker pulse sequence code | lcjresprqf | cosygpqfpr. Ht | dipsi2esgpp h | hsqcetgpprsisp 2.2.be | hmbcgplpndprqf |
| notes | tilted and symmetrised | t1 noise reduced |  |  |  |

**Supplementary Table 2: Typical data acquisition and processing parameters for 2D NMR spectra of plasma samples from an FMO5 knockout mouse at week 45**

| **Parameter** | **JRES** | **COSY** | **HSQC** |
| --- | --- | --- | --- |
| F2 spectral width (Hz) | 10,026 | 6,009 | 7,211 |
| F1 spectral width (Hz) | 78 | 6,009 | 24,875 |
| data points in t2 | 8,192 | 1,024 | 1,096 |
| spectral size in F2 | 8,192 | 2,048 | 2,048 |
| increments in t1 | 40 | 512 | 400 |
| spectral size in F1 | 512 | 2,048 | 2,048 |
| number of scans | 2 | 32 | 32 |
| relaxation delay (s) | 2.00 | 2.00 | 2.00 |
| apodisation | sine bell in t1 and t2 and first point correction in t1 | sine bell squared in t2, sine square with first point correction in t1 | sine bell squared in t2, sine square with first point correction in t1 |
| Bruker pulse sequence code | lcjresprqf | cosygpprgf | hsqcetgpprsisp2.2.be |
| other points t | tilted and symmetrised | t1 noise reduction applied |  |

**Supplementary Table 3: Full NMR Spectroscopic Data for Authentic 6-hydroxy-6-methyl-hexan-3-one and for the Metabolite in FMO5 KO urine (see Footnotes for structures and atom numbering)**

|  | **6-hydroxy-6-methyl-hexan-3-one in buffer/TSP (authentic sample ex Enamine)** | | | **6-hydroxy-6-methyl-hexan-3-one metabolite in week 30 FMO5 KO mouse urine with buffer/TSP** | | |
| --- | --- | --- | --- | --- | --- | --- |
|  |  |  |  |  |  |  |
| **Ketone** | **δ_H_ in ppm (multiplicity and coupling constants)** | **δ_C_ in ppm** | **additional 2D NMR connectivities** | **δ_H_ in ppm (multiplicity and coupling constants)** | **δ_C_ in ppm** | **additional 2D NMR connectivities** |
| 1 | 1.018 (t, 7.3 Hz) | 10.2 | 38.6 (C2), 222.0 (C3) | 1.015 (t, 7.3 Hz) | 10.1 | COSY to 2.598 (H2)  HMBC to 38.7 (C2) and 222.2 (C3) |
| 2 | 2.599 (q, 7.4 Hz) | 38.9 | 10.2 (C1), 222.1 (C3) | 2.598 (q, 7.3 Hz) | 38.6 |  |
| 3 | - | 222.0 | δ_C_ from HMBC | - | 222.3 |  |
| 4 | 2.618 (m, 2^nd^ order) | 40.1 | 39.2 (C5), 73.6 (C6) | 2.620 | 40.0 | HMBC to 39.2 (C5) and 222.4 (C3) |
| 5 | 1.740 (m, 2^nd^ order) | 39.4 | 30.4 (C6-Me, C7), 39.9 (C4), 73.6 (C6), 222.0 (C3) | 1.739 (m, 2^nd^ order) | 39.3 | COSY to 2.620  HMBC to 30.4 (C6-Me and C7), 39.9 (C4), 73.6 (C6) and 222.3 (C3) |
| 6 | - | 73.6 | δ_C_ from HMBC |  | 73.6 |  |
| 6-Me and 7 | 1.210 (s) | 30.5 | 30.4 (C6-Me and C7), 39.2 (C5) and 73.6 (C6) | 1.209 (s) | 30.4 | HMBC to 30.4 (C7), 39.2 (C5) and 73.6 (C6) |
| **Hemi-Ketal** | **δ_H_ in ppm (multiplicity and coupling constants)** | **δ_C_ in ppm** | **additional 2D NMR connectivities** | **δ_H_ in ppm (multiplicity and coupling constants)** | **δ_C_ in ppm** | **additional 2D NMR connectivities** |
| 1 | 0.932 (t, 7.5 Hz) | 11.0 | 35.5 (C2), 110.7 (C3) | 0.935 (t, 7.5 Hz) | N/O |  |
| 2 | 1.762 (m, difficult to analyse: resolution not optimal)  1.673 (m, ditto) | 35.7  35.5 | 11.1 (C1), 110.7 (C3)  11.1 (C1), 110.7 (C3) | N/O | N/O |  |
| 3 | - | 110.7 | δ_C_ from HMBC | - | N/O |  |
| 4 | 2.115 (d, d, d, 12.6, 10.8, 8.0 Hz)  1.920 (d, d, d, 12.5, 7.2, 2.6 Hz) | 37.8  37.9 | 39.2 (C5)  39.2 (C5), 86.2 (C6), 110.7 (C3) | N/O | N/O |  |
| 5 | 2.008 (m, difficult to analyse: resolution not optimal)  1.862 (m, ditto) | 39.2  39.3 | 30.1 (C6-Me), 31.9 (C7), 37.7 (C4), 86.1 (C6), 110.7 (C3, weak)  30.1 (C6-Me, weak), 110.7 (C3) | N/O | 39.0 |  |
| 6 | - | 86.1 | δ_C_ from HMBC | - | 86.2 | δ_C_ from HMBC |
| 6-Me | 1.200 (s) | 30.1 | 31.9 (C7), 86.1 (C6) | 1.197 |  | HMBC to 31.9 (C7) and 86.2 (C6) |
| 7 | 1.370 (q, 0.4 Hz) | 31.9 | 30.1 (C6-Me), 39.1 (C5), 86.1 (C6) | 1.367 |  | HMBC to 30.3 (weak, C6-Me), 39.0 (C5) and 86.2 (C6)  Weak COSY to 1.205 |

**Footnotes to Supplementary Table 3:**

1. multiplicities are: s, singlet; d, doublet; t, triplet; m, multiplet
2. N/O not observed
3. atom numbering for the ketone and hemi-ketal tautomers as in the structures below

**Supplementary Table 4: Experimental NMR Spectroscopic Data for the Statistically Significant Age-Discriminating Metabolites in the Urine of C57BL/6J Wild-Type and Flavin-containing Monooxygenase 5 (FMO5)-Knockout Mice**

**Supplementary Table 5: Experimental NMR Spectroscopic Data for the Statistically Significant Age-Discriminating Metabolites in the Plasma of C57BL/6J Wild-Type and Flavin-containing Monooxygenase 5 (FMO5)-Knockout Mice**
